# Patterns of Polymorphism and Demographic History in Natural Populations of *Arabidopsis lyrata*

**DOI:** 10.1371/journal.pone.0002411

**Published:** 2008-06-11

**Authors:** Jeffrey Ross-Ibarra, Stephen I. Wright, John Paul Foxe, Akira Kawabe, Leah DeRose-Wilson, Gesseca Gos, Deborah Charlesworth, Brandon S. Gaut

**Affiliations:** 1 Department of Ecology and Evolutionary Biology, University of California Irvine, Irvine, California, United States of America; 2 Department of Biology, York University, Toronto, Canada; 3 Institute of Evolutionary Biology, School of Biological Sciences, University of Edinburgh, Edinburgh, United Kingdom; Washington University School of Medicine in St. Louis, United States of America

## Abstract

**Background:**

Many of the processes affecting genetic diversity act on local populations. However, studies of plant nucleotide diversity have largely ignored local sampling, making it difficult to infer the demographic history of populations and to assess the importance of local adaptation. *Arabidopsis lyrata*, a self-incompatible, perennial species with a circumpolar distribution, is an excellent model system in which to study the roles of demographic history and local adaptation in patterning genetic variation.

**Principal Findings:**

We studied nucleotide diversity in six natural populations of *Arabidopsis lyrata*, using 77 loci sampled from 140 chromosomes. The six populations were highly differentiated, with a median FST of 0.52, and structure analysis revealed no evidence of admixed individuals. Average within-population diversity varied among populations, with the highest diversity found in a German population; this population harbors 3-fold higher levels of silent diversity than worldwide samples of *A. thaliana*. All *A. lyrata* populations also yielded positive values of Tajima's D. We estimated a demographic model for these populations, finding evidence of population divergence over the past 19,000 to 47,000 years involving non-equilibrium demographic events that reduced the effective size of most populations. Finally, we used the inferred demographic model to perform an initial test for local adaptation and identified several genes, including the flowering time gene FCA and a disease resistance locus, as candidates for local adaptation events.

**Conclusions:**

Our results underscore the importance of population-specific, non-equilibrium demographic processes in patterning diversity within *A. lyrata*. Moreover, our extensive dataset provides an important resource for future molecular population genetic studies of local adaptation in *A. lyrata*.

## Introduction

A thorough understanding of evolutionary history requires detailed information about both the genetic diversity underlying phenotypic variation and the forces that shape that diversity. Consequently, much effort is being devoted to identifying genes of functional significance and to assessing the relative importance of selection and demographic history in patterning genetic diversity. Both of these goals ultimately require genome-scale approaches. Even a simple phenotype may be the product of myriad genic interactions, and hence a genome-wide view may be necessary for a full understanding of the genetic components that contribute to a phenotypic trait. Similarly, study of genetic variation at one or a few loci is unlikely to be adequate for differentiating the effects of demography and selection, because patterns of diversity vary widely across the genome even under the simplest neutral equilibrium conditions. Non-equilibrium demographic processes can further increase this variance and mimic expected patterns of genetic diversity following selective events (reviewed by [1]). Large, multi-locus studies of patterns of genetic diversity have proven helpful for inferring the demographic histories of *Drosophila* [2] and humans [3], but, apart from *Arabidopsis thaliana* [4] and domesticated crops [5], such studies remain rare in plants.

To date, the few molecular population genomic analyses in plants have investigated variation at the species level, sampling one or few individuals from disparate locations across a species range without emphasis on local populations. Species-wide sampling is appropriate for testing for deviations from neutral equilibrium if metapopulation dynamics apply [6], and may also be suitable for inferring major demographic changes in a species' history [7]–[10]. However, species-wide samples are not suitable for investigating population processes of divergence, demographic change, and local adaptation. To understand the maintenance of variation within species, and the importance of local selection and demography in natural populations, both within- and among-population sampling are needed.

Despite the importance of local population processes to plant evolution, surprisingly little attention has been given to the distribution of DNA sequence diversity among plant populations. Although many studies have used genetic markers to study genetic differentiation among plant populations [11], [12], so far only a handful have examined DNA sequence diversity within and among natural plant populations [13]–[21]. Unlike other molecular marker systems, DNA sequence data provide information about recombination and linkage disequilibrium (LD), which can be highly sensitive to demographic history [22], [23]. Work done to date has demonstrated the need for local sampling to accurately describe patterns of LD, diversity, and the frequency spectrum of polymorphisms in local populations [13], [19], and shown that even simple demographic processes can better explain observed data than can the assumption of neutral equilibrium [15], [20]. Nonetheless, many of these studies have relied on small samples of loci or groups of candidate genes, neither of which is likely sufficient to capture patterns of genome-wide variation or provide insight into the relative roles of demographic history and selection.

Here we present a large-scale population-genetic analysis of sequence diversity in natural populations of *Arabidopsis lyrata. A. lyrata* is a predominantly self-incompatible, perennial species with a circumpolar distribution across northern and central Europe, Asia, and North America. *A. lyrata* appears to maintain large, stable populations, particularly in Central Europe, where populations are hypothesized to have served as refugia during the most recent Ice Age [24], [25]. *A. lyrata* has become a model system for plant molecular population genetics [26]–[29] and for investigating local adaptation. For example, divergent selection on trichome production has been found among phenotypically differentiated Swedish *A. lyrata* populations [30], [31]. Flowering time and floral display also appear to be under strong selection, with large differences in day-length requirements between Northern and Southern populations [32], [33]. *A. lyrata* is also of great interest because it is a close relative of *A. thaliana* [24], [26]–[29], providing opportunities for comparative studies of the consequences of differences in breeding system [34], [35], demographic history [36], and selection [37], [38].

**Figure 1:**
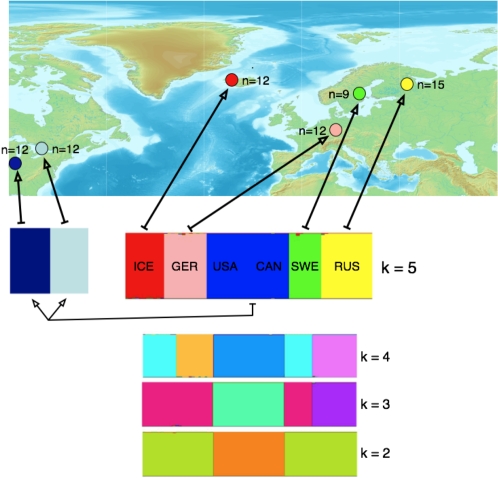
A map showing the locations and sample size (N) for the 60 long exons for each of the six populations studied: ICE = Iceland, GER = Germany, CAN = Canada; SWE = Sweden; RUS = Russia. The results of structure analyses are shown below the map. The mostly likely number of clusters (*k*) is five. Considered separately, the USA/Canada individuals clearly differentiate into two geographically separate clusters.

We survey diversity at 77 gene fragments sampled from multiple plants from each of six natural *A. lyrata* populations. The six populations are located in Germany, Russia, Sweden, Iceland, the United States, and Canada (Fig. 1), representing much of the geographic range of diploid populations of the species. Our first objectives with this large resequencing dataset are to quantify patterns of sequence diversity within and among *A. lyrata* populations. We then employ information about levels and patterns of diversity to model aspects of the demographic history of *A. lyrata* populations. We demonstrate that our models capture many of the important features of the observed genetic variation, and then use this information about demographic history to make preliminary searches for signals of local adaptation. Finally, we contrast patterns of diversity in *A. lyrata* to previously published information about diversity in *A. thaliana*.

## Materials and Methods

### Plant materials and DNA sequencing

We utilized seed collected from six natural populations of *A. lyrata*, representing both subspecies and much of the geographic range of diploid populations (Fig. 1). Seeds representing *A. lyrata* ssp. *petraea* were collected in Plech, Germany (by M. Clauss); a location near Reykjavik, Iceland (by E. Thorhallsdottir); and Karhumäki, Russia and Stubbsand, Sweden (both by O. Savolainen). Two North American locations - Rondeau Provincial Park, Ontario, Canada and Indiana Dunes, Indiana, U.S.A. (both provided by B. Mable) – were the source for *A. lyrata* ssp. *lyrata* seeds. Once germinated, juvenile plants were harvested, and genomic DNA was extracted using the DNeasy plant mini kit.

We amplified and sequenced a set of 60 large exons, plus 17 gene fragments including introns. All loci are from chromosome arm regions, excluding pericentromeric region genes where close linkage to other loci could affect their diversity [28]. As explained in [28], the large exons were chosen by length, without respect to function, targeting exons up to 800 bp in the *A. thaliana* genome sequence. Each exon was used as a BLAST query against the shotgun genome sequence of *Brassica oleracea*. Homologous *B. oleracea* regions were aligned to *A. thaliana,* and PCR primers were designed to conserved regions, using PrimerQuest (Integrated DNA Technologies). The single-copy status of primers was ensured by another round of BLAST queries to the *A. thaliana* genome. The 60 long exons were sequenced in a sample of 71 plants: 12 from Germany, 12 from Iceland, 12 from Canada, 11 from the USA, 9 from Sweden, and 15 from Russia. The 17 intron-containing gene fragments were amplified in a total of 32 individuals from the same six populations: seven from Iceland, five from Germany, five from Canada, five from the USA, five from Russia, and five from Sweden. A list of loci, the number of alleles sampled, and the gene ontology terms for each locus is available in Table S1.

PCR amplifications were based on conditions that included 30 cycles of 30 seconds denaturing at 95°C, 45 seconds annealing at 55°C, and 60 seconds extension at 70°C. Amplification products were sequenced directly on both strands using ABI BigDye 3.1 and the ABI 3100 automated sequencer, but gene fragments were cloned and sequenced when samples were found to be heterozygous for indels. Bases were called using Sequencher v. 4.1, using the ‘call secondary peaks’ option to aid in the identification of heterozygous sites. All putative heterozygous sites were checked manually, and only data that could be confirmed on both strands were included in the analysis. Sequence data were submitted to Genbank (BV683158-BV686427; EF502173-EF502282; EF502359-EF502483; EF502558-EF502973).

### Sequence statistics and analysis

Standard diversity statistics, including nucleotide diversity θ_π_, Watterson's [39] estimator θ_w_, numbers of segregating sites S, numbers of segregating sites shared between populations and unique to individual populations, and Tajima's D [40] were calculated from biallelic silent (noncoding and synonymous) sites using a modified version of the analysis package of software from the libsequence C++ library [41]. LD and recombination were also estimated from silent sites. LD was analyzed with the squared correlation coefficient, r^2^, using Weir's method for estimating linkage disequilibrium from unphased diploid data [42]; R code was provided by S. Macdonald (U. Kansas). The population recombination parameter ρ was estimated using the LDHAT program [43]. Values of ρ were estimated only for loci with S ≥ 5, due to the poor performance of estimators when there are few segregating sites [44]. Among-population differentiation for each locus was estimated using F_ST_, calculated as 1-π_S_/π_T_, where π_S_ is the average within population nucleotide diversity weighted by the sample size of the locus, and π_T_ is the total nucleotide diversity of the pooled sample across populations.

We used the software PHASE 2.1 [45], [46] to generate haplotype data from all SNPs in the data for Bayesian cluster (structure) analysis. We assumed that the entire sample originated from a single random mating population, to avoid biasing the structure analysis. Haplotypes were reconstructed for 55 genes from the large exon data set (five loci were excluded due to computational difficulties associated with high recombination and polymorphism), and those with the highest posterior probabilities were used in cluster analysis performed with the program structure 2.1.1 [47]. The program was run assuming values of *k* (population number) from 2 to 7, each with 100,000 repetitions, and a burn-in period of 10,000. Similar results were obtained from multiple structure runs; we report results only from the run with the highest overall likelihood.

### Demographic modeling

We employed a Bayesian approach to estimate demographic parameters. Briefly, we simulated data under a specified demographic model, drawing parameters of the model from designated prior distributions. Summary statistics were calculated for each simulated data set and compared to values from the observed data; simulations with summary statistics that best approximated the observed summary statistics informed the posterior distributions for each parameter and formed the basis for parameter inference. We limited our demographic inference to pairwise models of divergence between two populations in order to avoid the computational difficulties of simultaneously estimating numerous parameters. Because our diversity data concur with previous work that has cast Germany as a center of *A. lyrata* diversity and a potential refugium [24], we used our German sample as a reference to compare to each of the remaining five populations in pairwise fashion.

**Figure 2:**
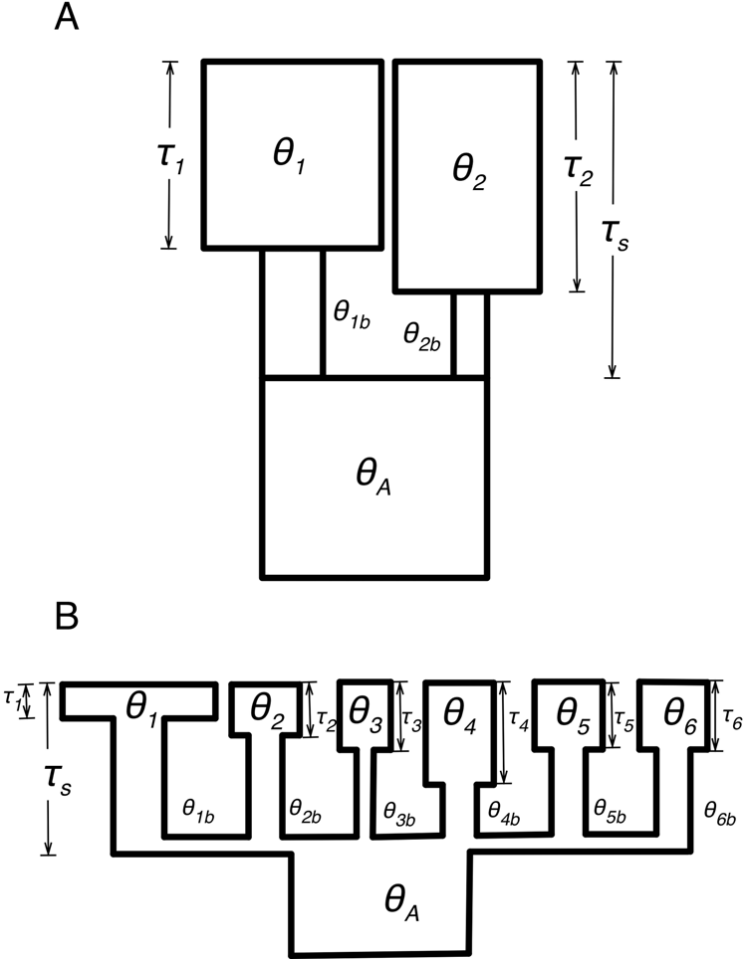
Demographic models. A) Schematic representation of the two-population bottleneck model used for parameter estimation. B) Schematic of the six-population model used for testing single-locus fit of F_ST_. See text for parameter descriptions.

Our demographic model (Figure 2A) posits an ancestral population, which splits into two daughter populations that each experience population bottlenecks. The ancestral population evolves with a population mutation rate θ_A_ = 4Ne_A_μ, where Ne_A_ is the ancestral effective population size and μ is the per nucleotide neutral mutation rate. At time τ_s_ generations in the past, the ancestral population splits into two bottlenecked ‘founding’ populations of size θ_1b_ and θ_2b_. The daughter populations remain small until they recover from their bottlenecks to modern sizes of θ_1_ and θ_2_ at times τ_1_ and τ_2_ in the past. In total, we estimated eight parameters: the population mutation rate θ_1_ of our reference Germany population, the ratios θ_2_/θ_1_, θ_A_/θ_1_, θ_2b_/θ_1_, θ_1b_/θ_1_, the divergence time τ_s_, and the times since recovery from the bottlenecks, τ_1_ and τ_2_. Recent estimates of the recombination rate in *A. lyrata* [48], [49] yield values very close to estimates of the substitution rate [50], and we therefore assumed that the population recombination rate ρ_Α_ = 4Ne_A_r is identical to θ_A_. We further assumed that both μ and r, the per nucleotide recombination rate at neutral sites, are invariable across populations (but vary among loci).

Our simulations drew from prior distributions of each of the parameters to be estimated. For the prior distribution of θ_1_ we fitted a gamma distribution to the observed values of θ_w_ at silent sites across all 77 genes from the German population. Prior distributions for θ_2_/θ_1_ and θ_A_/θ_1_ were uniform on 0–1 and 0.5–2, respectively, while θ_2b_/θ_1_ and θ_1b_/θ_1_ were drawn from uniform distributions on the intervals of 0-θ_2_ and 0-θ_1_. For the divergence time τ_S_, we used a log-uniform prior, thus assigning substantial probability to recent post-glaciation divergence while still allowing for more ancient divergent times. We sampled τ_S_ from the interval 0.0001–0.075, roughly translating to 100–90,000 years. Values of τ_1_ and τ_2_ were then drawn from a log-uniform distribution on the interval 0.0001-τ_S_. For each pairwise comparison we simulated 5 million multilocus datasets for a total of ∼2.3 billion coalescent simulations. All datasets were simulated using the observed sample sizes and length (in silent sites) for each locus.

To summarize the data, we made use of an array of statistics widely utilized for demographic inference (e.g. [51]–[53]): F_ST_ and the number of shared segregating sites S_S_ between populations, and S and θ_π_ for each population. We calculated the mean and variance of each statistic for each simulated dataset, and estimated the posterior probability distribution of the eight model parameters following the regression approach of Beaumont *et al*. [54,54]. Summary statistics were transformed following Hamilton *et al.* [55] prior to the regression, and acceptance values of 10^−3^ were used for all analyses. We utilized a modified version of the ms program [56] to perform coalescent simulations; ms command lines are in Text S1. Regression analysis made use of code provided by K. Thornton (www.molpopgen.org).

## Results

### Levels and Patterns of diversity

**Figure 3:**
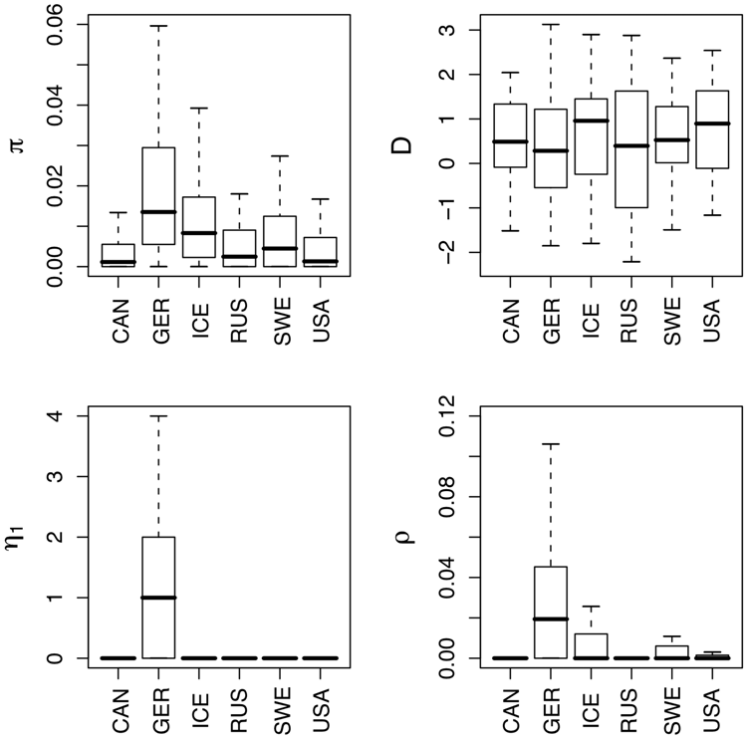
Silent site diversity within populations. A) Shown are boxplots of θ_π_, Tajima's D, the number of singletons η_1_ and the population recombination rate ρ for each population. Bars represent the median, boxes the interquartile range, and whiskers extend to 1.5-times the interquartile range.

We sequenced an average of 95 alleles per locus for 77 loci in plants sampled from 6 natural populations of *A. lyrata* from across the range of the species (Fig. 1). The sequences average ∼530bp in length, yielding a total of more than 3.75 Mb of aligned sequence. Mean silent site nucleotide diversity (θ_π_) for the entire dataset is 0.0225, but diversity differs greatly among the six populations (Fig. 3). The mean and median θ_π_ at silent sites in the German sample are 0.0135 and 0.0209, respectively, substantially higher than for the other five *A. lyrata* populations. The Icelandic (0.0129 mean, 0.0083 median), Swedish (0.0097 mean, 0.0045 median), and Russian (0.0071 mean, 0.0025 median) samples have intermediate diversity levels, and the U.S. (0.0060 mean, 0.0013 median) and Canadian (0.0055 mean, 0.0012 median) samples have the lowest diversity.

Tajima's D, a measure of the skew of the site frequency spectrum, is positive at silent sites in all populations, indicating a general paucity of low-frequency polymorphisms, but it is closest to the neutral expectation of approximately zero in the German sample (median D = 0.28; Fig. 3). Comparison of the number of singletons (η_1_) at silent sites among populations reveals a similar pattern. Although most populations have at least a few loci with η_1_>0, the median value of η_1_ per gene differs from zero only in the German population (Fig. 3).

The population recombination parameter, ρ, can also provide insights into recent population history [22]. Because we estimated ρ only for loci with S≥5 at silent sites, estimates are available for only a subset of loci in each population (Table S2). Nonetheless, differences in ρ among populations are even more pronounced than nucleotide diversity differences (Fig. 3). While the median estimate of ρ per silent site in Germany, 0.0194, is close to the estimate of θ_π_, the median estimate for all other populations is 0. Intra-locus LD at silent sites is consistent with the estimates of ρ: median r^2^ values are lowest in Germany (0.29), followed by Iceland (0.59), Canada (0.62), Sweden (0.63), USA (0.66) and finally Russia (1.0). LD also decays most rapidly with distance in the German population (Figure S1).

**Figure 4:**
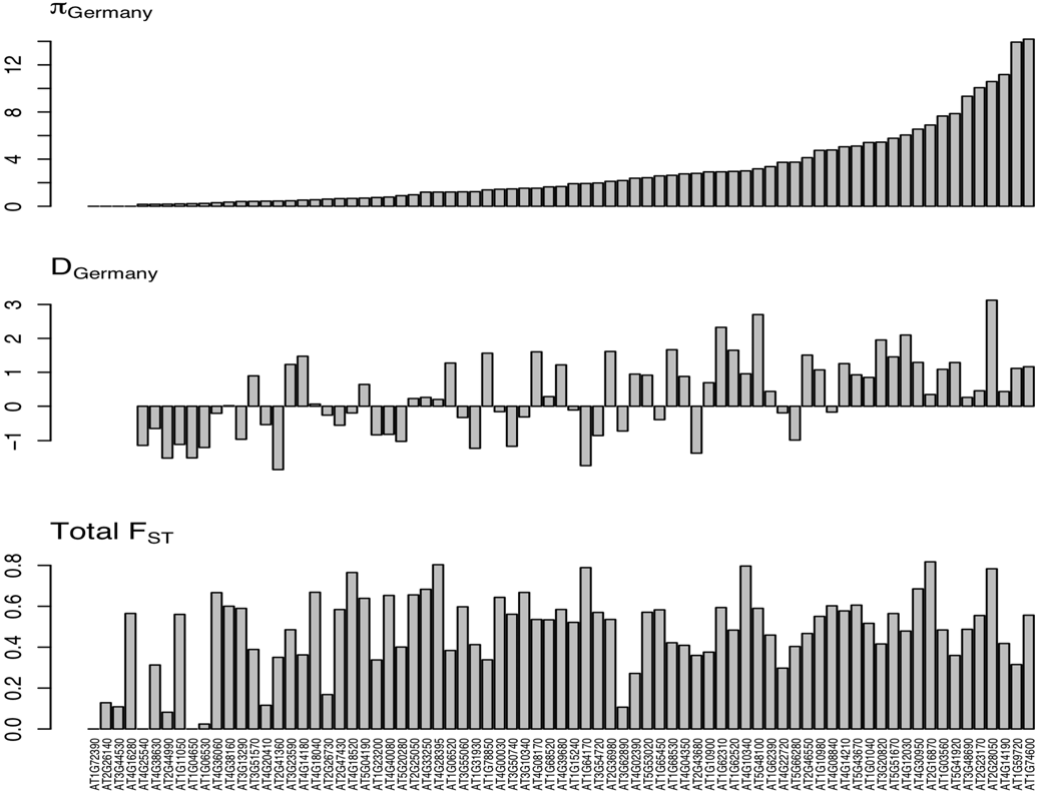
Summary statistics at silent sites among 77 loci in the German population. Shown are θ_π_ per base pair, Tajima's D and range-wide F_ST_. Loci are shown in the same order as in Supplementary Tables S1 and S2.

Differences in patterns of diversity are apparent among loci as well as among populations. Among-locus variation is shown for the German population in Fig. 4; summary statistics for other populations can be found in Table S2. Values of silent site nucleotide diversity (θ_π_) in the German population range from zero (at four loci) to 0.099 per bp at locus AT1G74600. Tajima's D varies from −1.85 to 3.12, with a mean (0.32) significantly different from zero (p<0.02, t-test, 72 df). The distribution of ρ per silent site, based on the 42 loci with S≥5, is strongly leptokurtic, with estimates ranging from ρ  = 0 at 14 loci to extremes of ρ>0.9 at loci AT3G10340 and AT1G65450.

Three loci contain polymorphic stop codons. Two premature stop codons are present in the leucine-rich-repeat (LRR) gene AT3G51570; both stop codons are polymorphic in the Iceland population but fixed in the US, Canada, and Sweden. Locus AT3G20820, also an LRR gene, is polymorphic for a single stop codon in both Sweden and Germany, and AT1G10900, a gene coding for a phosphatidylinositol-4-phosphate 5-kinase family protein, is segregating for a stop codon in both Iceland and Germany. Polymorphic stop codons are frequent in LRR genes and may play a role in balancing selection for disease resistance [57], but we are unaware of previous evidence that might explain a similar pattern at locus AT1G10900.

### Population structure

Estimates of F_ST_ and a structure analysis suggest long-term geographic subdivision among the populations studied. Among all populations, the median F_ST_ across loci at silent sites is 0.52, with per-locus values ranging from 0 to 0.82 (Fig. 4). Similarly, structure results from analysis of all SNPs in the data identify a most likely model of *k* = 5, with all individuals clustering according to their population of origin, except that subspecies *lyrata* individuals from North America group as a single cluster. Separate analysis of the two North American populations nonetheless reveals *k* = 2 as the most likely number of clusters, and population assignments coincide perfectly with geographic locations (Figure 1). Neither structure analysis provides evidence for recent admixture in any of our samples. High levels of population structure coupled with an absence of genetic admixture paint a picture of long-term geographic subdivision among our populations, offering no evidence of ongoing or recent migration.

**Figure 5:**
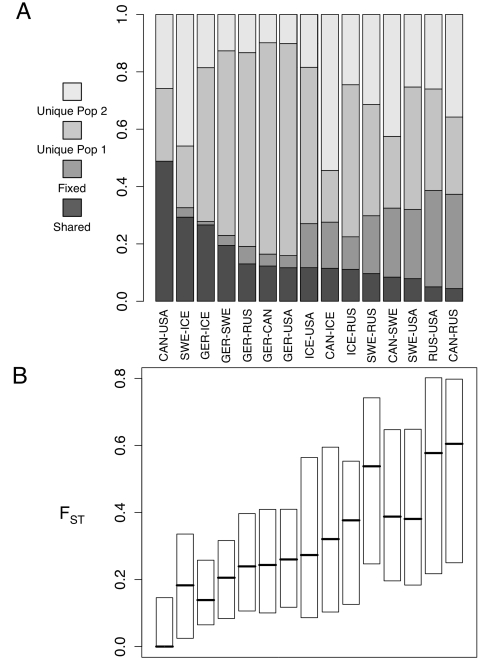
Pairwise population differentiation at silent sites. A) shared and unique polymorphisms and fixed differences. B) F_ST_. Boxplots show the median and interquartile distance of values across loci.

Additional insights into population structure were obtained from analysis of variants shared between pairs of populations, which further suggest strong population differentiation. With the exception of the two ssp. *lyrata* populations, shared variation makes up less than 30% of the SNP variants in any pairwise comparison of populations (Fig. 5A). Strikingly however, five of the seven comparisons with the highest percentages of shared variants involve the German population. In general, pairwise comparisons involving the German sample show low percentages of fixed variants and low pairwise F_ST_ values (Fig. 5B). The German sample also has a higher proportion of unique (private) variants than other populations, consistent with its higher overall diversity. Also of note are the high proportion of fixed differences (∼33%), high F_ST_, and low shared variation between the Russian and both N. American populations, results which suggests that their coalescent histories are nearly independent.

### Demographic history

The observation of positive Tajima's D values, low diversity, and high LD in most of our populations suggests a history of population bottlenecks. Building on these observed patterns of diversity and structure, we used Bayesian inference methods [54] to infer features of the demographic history of these populations. We restricted our parameter inference to two-population models (Fig. 2A) to avoid unmanageable numbers of parameters in a full six-population model. For these two-population models, we treated the German population as a reference population with which we compared each of the remaining five populations in pairwise fashion. The use of the German population as a reference seems justified by previously published analyses showing the high diversity of Central European populations and suggesting that these populations may represent refugia [24], [25], as well as by our own data that show the German sample had higher diversity, less skew of the frequency spectrum, and less LD than other populations. Nonetheless, positive values of Tajima's D (Fig. 3) suggest that even the German sample is not at equilibrium; we thus chose to model population bottlenecks for both diverging populations in each comparison. High F_ST_ and a lack of evidence for admixture in our cluster analyses also led us to model divergence in isolation. Further justification for this choice comes from explicit estimation of migration rates in an independent demographic model that yielded no evidence for substantial introgression (the number of migrants per generation was estimated to be ≪1 for all pairwise comparisons; data not shown).

**Figure 6:**
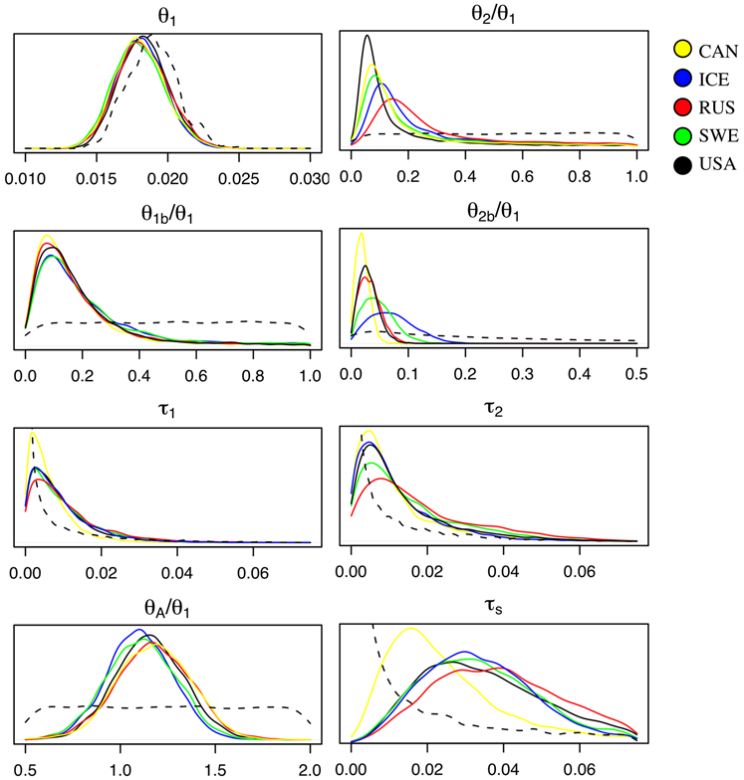
Posterior distributions for the parameters of the pairwise population divergence models. Dashed curves represent the Bayesian prior for each parameter. Point estimates of the parameters for each population are shown in Table 1.

We estimated the posterior probability distribution of each of the parameters of our divergence models (Table 1, Fig. 6), comparing the German population to the other five populations in pairwise fashion. Each of the posterior distributions is contained well within the range of its specified prior distribution (Fig. 6), thus providing some assurance that we adequately sampled the parameter space. Assuming a mutation rate of 1.5×10^−8^ per year [50] and a generation time of 2 years, parameter estimates of the model can be converted into estimates of effective population size (N_e_) and time in years (Table 1). All five pairwise models are in close agreement on the effective population size of the reference German population (∼75,000 individuals) and the size of the bottleneck for this population (∼7,000 individuals). The estimated ancestral effective population size is ∼86,000, suggesting a nearly complete post-bottleneck recovery in the German population. For three populations (Sweden, Iceland and USA), the estimated divergence times from the German population are quite similar, ∼35,000 years. The point estimate for the Russian population is older (∼47,000) but the distribution of τ_S_ is similar to that for Sweden, Iceland and USA. The divergence estimated for the Canadian population is more recent (∼19,000 years) than the other four populations. Finally, the posterior distributions for the recovery times (τ_1_ and τ_2_) are not strongly differentiated from their prior distributions (Fig. 6), suggesting that our model estimates these parameters poorly.

**Table 1:** Parameter estimates for the demographic model (Fig. 5–6).

	ICE	USA	CAN	RUS	SWE
θ_1_	0.0182	0.0182	0.0177	0.0179	0.0176
	76000	76000	74000	74000	73000
θ_1b_	0.0903	0.0964	0.0753	0.0754	0.0947
	7000	7000	6000	6000	7000
τ_1_	0.0027	0.0025	0.0018	0.0035	0.0026
	3000	3000	2000	4000	3000
θ_A_	1.0994	1.1555	1.1915	1.1666	1.1250
	83000	88000	88000	87000	83000
θ_2_	0.1064	0.0565	0.0716	0.1430	0.0818
	8000	4000	5000	11000	6000
θ_2b_	0.0576	0.0246	0.0183	0.0240	0.0389
	4000	2000	1000	2000	3000
τ_2_	0.0047	0.0051	0.0047	0.0078	0.0052
	6000	6000	6000	9000	6000
τ_S_	0.0297	0.0267	0.0157	0.0390	0.0310
	36000	32000	19000	47000	36000

Point estimates of each parameter in each population are listed. Below the point estimates are values converted to N_e_ (for θ) and years (for τ). Conversion to N_e_ assumes a generation time of 2 years, and both N_e_ and divergence time conversions assume a neutral mutation rate of 1.5×10^−8^ per site per year.

We tested the fit of our estimated model to the data, using the ‘predictive posterior’ approach [58]. Using locus-specific observed values of θ_w_ at silent sites from our German population, we drew parameter values from the estimated posterior distributions of our fitted model and simulated 10,000 multilocus datasets, comparing the distribution of summary statistics from these simulations to our observed data (Table 2). The model provides a remarkably good fit to the data: the observed mean and variance of all summary statistics are well within the central 95% credible interval of the simulated data. Although we used no summaries of the site frequency spectrum other than θ_π_ in our model estimation, the observed Tajima's D values in non-German populations fit nearly as well as the statistics used in the model estimation. Tajima's D fits equally well in Germany (data not shown) but is not independent of θ_π_ because θ_w_ was fixed for these simulations. The ability to fit Tajima's D contrasts markedly with other studies that have inferred demographic history by similar methods (e.g. [51]). These results generally suggest that our model successfully captures many of the important features of our data.

**Table 2:** Predictive posterior results from 10,000 coalescent simulations under the estimated pairwise demographic model.

Pop.	F_ST_	S_S_	S_1_	θ_π1_	S_2_	θ_π2_		D_2_
MEAN								
RUS	0.08–0.60	0–5.4	2.5–11.2	0.8–3.8	0.6–8.8		0.2–3.1	−0.60–0.77
	0.29	1.3	8.1	3	2.7		0.9	0.37
SWE	0.09–0.53	0.1–4.6	2.9–11.1	1.0–3.8	0.8–6.9		0.3–2.6	−0.26–0.78
	0.22	1.9	8.1	3	3.2		1.2	0.54
ICE	0.10–0.48	0.3–4.6	2.7–10.6	0.9–3.6	1.4–7.1		0.5–2.6	−0.1–0.81
	0.17	2.6	7.9	2.9	4.6		1.8	0.71
USA	0.14–0.59	0–3.5	2.8–10.8	0.9–3.7	0.3–5.2		0.1–2.0	−0.51–0.77
	0.28	1.3	7.9	2.9	2.1		0.9	0.77
CAN	0.05–0.57	0–6.2	2.7–11.5	0.9–3.9	0.4–9.0		0.1–3.1	−0.53–0.84
	0.28	1.1	7.9	2.9	2.1		0.8	0.4
VARIANCE								
RUS	0.01–0.11	0–41.1	12.7–137.5	1.8–19.5	1.0–88.9		0.1–13.7	0.73–2.12
	0.05	7.3	62.3	11.1	13.3		1.7	2.32
SWE	0.01–0.11	0.3–38.7	14.9–133.4	2.2–19.0	2.3–71.1		0.3–12.1	0.87–2.01
	0.03	10	62.3	11.1	15.5		2.2	1.04
ICE	0.01–0.10	0.8–39.5	13.5–122.3	2.0–17.5	6.0–75.8		0.8–11.7	0.91–1.94
	0.03	12	59.9	10.6	33.3		5.4	1.23
USA	0.02–0.11	0–28.7	13.7–128.5	2.0–18.4	0.5–50.9		0.1–8.9	0.77–2.11
	0.05	7	59.1	10.5	16.7		3.9	1.26
CAN	0–0.11	0–52.06	13.0–144.1	1.9–20.4	0.5–89.0		0–13.7	0.7–2.14
	0.05	10	59.9	10.6	23.1		3.9	1.13

The 95% credible interval of the multilocus mean and variance of summary statistics is given for each population; observed values of each summary statistic are given below. Statistics with the subscript 1 refer to the German population; those with subscript 2 refer to the listed population. Values in bold are significantly different from expectations under the model at p<0.05.

### Potential signatures of local adaptation in *A. lyrata*

To what extent are patterns of genetic diversity at individual loci associated with local adaptation? To begin to address this question, we performed simulations utilizing our inferred demographic model to generate expectations for individual loci under neutrality. For these initial tests, we selected F_ST_ as a measure because of its long history as an informative metric of local adaptation [59]–[61]. We first used the five demographic models inferred from the pairwise inter-population comparisons to generate neutral distributions of expected F_ST_ for silent sites and for all sites (including nonsynonymous variants). For each locus and model, we calculated F_ST_ from 10,000 single locus coalescent simulations drawn from our estimated posterior distributions. All simulations used the relevant length and observed θ_w_ (at silent or all sites) for each locus.

Our pairwise model only allows identification of loci that are extremely divergent between Germany and other populations, but cannot identify loci that reveal evidence of local adaptation among non-German populations. In a first attempt to identify such loci., we used our demographic machinery to build a model that includes all six populations explicitly. To build this model, we averaged the distributions of the parameters θ_1_, θ_1b_, τ_1,_ θ_A_, and τ_S_ across all five pairwise models to build a model in which all six populations diverge simultaneously from a common ancestor, but each undergoes an independent population bottleneck and recovery (Fig. 2B). A simple test of this combined model suggests it is not unreasonable: silent site F_ST_ across all populations simulated under the model fits well with the observed mean (observed 0.47, simulated 95% CI 0.39–0.59) and variance (observed 0.040, simulated 95% CI 0.028–0.057) of F_ST_ in the data.

Six loci (∼8%) deviate from the range of F_ST_ values expected under our neutral null models (Table 3). Pairwise comparisons identify four loci as outliers: in Russia locus AT2G26140, a gene in the UDP-glucosyl transferase family; in Canada both AT4G16280 (the flowering time locus FCA), and AT3G51570, an LRR disease resistance gene; and in Iceland and Sweden locus AT1G15240, a phox domain-containing protein. The 6-population model highlights two genes with unusually high F_ST_ across all populations: locus AT1G74600, a pentatricopeptide (PPR) containing gene, and AT5G53020, an unknown expressed protein. Only the FCA locus remains statistically significant after correction for multiple tests.

**Table 3:** Candidate loci for local adaptation.

	Pairwise		All Pops		
Locus	Silent	All Sites	Silent	All Sites	GO Terms
AT1G15240	–	ICE, SWE	–	YES	phox (PX) domain-containing protein
AT1G74600	–	–	–	YES	pentatricopeptide (PPR) repeat-containing
AT3G50740	RUS	–	–	–	UDP-glucoronosyl/UDP-glucosyl transferase family
AT3G51570	CAN	CAN	–	–	disease resistance protein (TIR-NBS-LRR class)
AT4G16280	CAN	–	–	–	flowering time control protein/FCA gamma
AT5G53020	–	–	–	YES	expressed protein

Shown are loci with values of F_ST_ which reject the null demographic model at p<0.05. For each locus, the table lists the populations which reject the null pairwise model and whether or not it rejects the 6-population model.

## Discussion

We present here two significant advances towards understanding sequence diversity in natural plant populations. The first is simply a much larger data set than in most studies of sequence diversity of natural plant populations, including explicit and extensive sampling both within and among populations. Second, we used demographic modeling, which, to date, has mostly focused on humans (e.g., [52]) and *Drosophila* (e.g., [58], [62]). Explicit modeling of natural population history remains rare in studies of flowering plants, though it has been applied to studies of cultivated plants [5], [63] and to a lesser extent, conifers [15], [20]. Our efforts permit parameter estimation for biologically meaningful demographic models and provide a direct measure of our confidence in the model and its relevance to our data.

### Diversity and population history

Our results build on previous work that documents high differentiation among *A. lyrata* populations [24], [29], [64], [65], and points to central European populations as a center of diversity for *A. lyrata* ssp. *petraea* [24], [25]. Other studies have further argued that central European populations may have served as refugia from which Northern Europe was re-colonized after glacial cycles during the Pleistocene [36], and even specifically hypothesized that the Icelandic population of *A*. *lyrata* ssp. *petraea* and North American populations of *A. lyrata* ssp. *lyrata* were colonized from Europe [26], [29].

Our results broadly concur with these ideas. Relative to the Central European (Germany) population surveyed here, other populations reveal the hallmarks of population bottlenecks: lower diversity, loss of singleton and low frequency variants, higher LD and lower estimated ρ values. The demographic inferences summarized in Table 1 suggest strong bottlenecks with little subsequent recovery of size in the non-German populations. Moreover, although most loci show strong genetic structure (median pairwise F_ST_ at silent sites among all populations = 0.52), differentiation is lower with the German population (median pairwise F_ST_ = 0.21). Pairwise comparisons also reveal a high proportion of shared variants and few fixed differences between Germany and other populations. Even populations as different genetically and geographically as Canada and Russia each possess extensive shared variation with Germany, suggesting that the non-German populations sampled represent subsets of the diversity in Germany. Consistent with this, all of our pairwise comparisons show a higher proportion of unique variants in Germany.

Both F_ST_ and Bayesian cluster analyses reveal unusually strong population structure for an outcrossing herbaceous species [11], providing little evidence for recent admixture or gene flow, but suggesting long-term persistence of isolated populations. This finding is supported by analysis of an alternate demographic model that explicitly estimated low pairwise migration between Germany and other populations (data not shown). It is possible, of course, that migration from unsampled populations or species contributes to observed patterns of diversity. One would expect such migration to increase both diversity and LD, but our data show higher LD only in non-German populations with lower levels of diversity. Although the data to explicitly test this hypothesis are not currently available, our sequence data provide no compelling evidence that migration from unsampled populations has strongly affected our sampled populations.

Although our demographic model does not aim to infer a definitive history, it is important to consider how inclusion of non-equilibrium processes may affect estimation of divergence times. Our estimates (Table 1) are much lower than calculations based solely on median pairwise F_ST_ values [66], which yields divergence times ranging from ∼90,000 years between Germany and Iceland to ∼170,000 years between Germany and Russia. However, our estimates are considerably older than the end of the most recent Ice Age, when Northern Europe was most likely re-colonized by *A. lyrata* [36]. We note, however, that the 95% credible intervals of our estimates generally include times as recent as 10,000 years ago, and that because τ_S_ estimates in years are proportional to the mutation rate, a rate twice as high as that estimated by Koch *et al.* [50] would reduce the value in years of our divergence time estimates by half. Alternatively, both the observed strong genetic structure and deep divergence time estimates are consistent with the possibility that populations of *A. lyrata* ssp. *lyrata* have persisted throughout the last glacial period [25].

### Local adaptation

The existence of large, ecologically stable populations in *A. lyrata* makes it suitable for studying local adaptation. Indeed, a growing number of *A. lyrata* genes show evidence of local adaptation. Examples include the trypsin inhibitor *ATTI2* gene in the Plech, Germany population [37], a centromeric region in the Russian population [28], a centromere specific histone gene [67], and a gene for trichome density in Swedish populations [30].

Building on the idea that locally adapted loci should have increased differentiation relative to neutral loci [61], we identified genes that exhibited extreme values of F_ST_ compared to expectations under the estimated demographic null model. This approach has the advantage of explicitly incorporating divergence and demographic processes, which can otherwise confound assessment of selection. Simple scans for loci of unusually low diversity would incorrectly suggest selection at many of the loci surveyed: in the Canadian population, for example, nearly half of the loci sequenced are devoid of variation (Table S2). However, like tests of the site frequency spectrum [68], diversity [69], or LD [70], our approach can only reject a neutral null model – it cannot provide evidence in favor of an alternative model with selection. It will also be limited by the accuracy and appropriateness of the demographic model used. However, our inferred model fits the data well (Table 2), including a measure of the site frequency spectrum (Tajima's D) that was not used to fit the model.

One difficulty with our model-based approach is that its statistical power is not well known. Because the initial demographic model is fitted to summaries of all the data from all loci, including variances of summary statistics across loci, it accounts for the full range of polymorphism among loci, including loci affected by recent selection. Simulating from the posterior distribution of parameter values rather than point estimates similarly broadens the range of summary statistics produced. The results of this analysis are thus likely to be conservative, sacrificing some power to detect selection but minimizing the potential for false positives. Finally, we have focused exclusively on F_ST_ as a measure of selection. While this is appropriate for our interest in local adaptation, it may well miss loci affected by other forms of selection, such as balancing selection or species-wide selective sweeps.

Six of our 77 genes yield evidence of deviation from the demographic model by this approach (Table 3). Although only one locus, AT4G16280, remains statistically significant after controlling for multiple tests, several of these loci deserve special attention, and all of them should be considered candidate genes that require further examination in the future. The one remaining significant locus encodes the flowering time control protein FCA and has only a single fixed noncoding site that differentiates Germany from Canada. The locus is nonetheless notable because there are no segregating silent sites in either Germany or Canada and there are no fixed differences in any other population at this locus. Intriguingly, the single noncoding site occurs as part of a repeat in intron 13, an intron in which alternative splicing creates a putatively nonfunctional FCA transcript [71]. Although FCA has not been previously implicated in local adaptation, it plays an important role in the flowering time pathway in *A. thaliana* [71], [72], and is thought to be important in adaptation to new flowering regimes [73]. Furthermore, a QTL for local adaptation of flowering time maps near the *A*. *thaliana* FCA locus [74], suggesting that our inference is biologically plausible.

Members of the LRR disease resistance and PPR-containing gene families warrant special mention as well. One LRR gene, locus AT3G51570, is identified as an outlier in the Canadian population and has the highest pairwise F_ST_ of any locus in the US population. Moreover, both North American populations are fixed for two premature stop codons at AT3G51570, and polymorphic stop codons are found both at this locus and a second LRR gene, locus AT3G20820. Locus AT1G74600, a PPR containing gene, is identified as an outlier by the six-population model and has nearly 30 fixed differences between subspecies *lyrata* and *petraea*. In fact, PPR genes as a group show interesting patterns of variation, exhibiting the four highest values of θ_π_ at silent sites in Germany and the four highest range-wide values of F_ST_ for silent sites. Some PPR genes play a role in cytoplasmic male sterility, and, like LRR genes, may be subjected to arms-race evolutionary dynamics driven by local adaptation events [75]–[78]. Finally, it is important to note that in addition to allowing for initial tests for loci important to local adaptation, our data provide baseline information on patterns of polymorphism and demographic history that should serve as a valuable resource for future studies of selection in *A. lyrata*.

### Contrasting polymorphism in *A. lyrata* and *A. thaliana*

In addition to facilitating inferences about *A. lyrata* population history, our extensive data provide an excellent opportunity to compare levels and patterns of diversity between *A. lyrata* and its close congener *A. thaliana*. Diversity in *A. lyrata* and *A. thaliana* differs in at least three ways. First, diversity in *A. lyrata* is higher than in *A. thaliana*, both within populations and species-wide [79], [80]. Nucleotide diversity at silent sites for our pooled dataset, for example, is nearly three times higher than diversity at synonymous sites in a world-wide *A. thaliana* sample [79], and many local populations of *A. thaliana* lack genetic diversity almost entirely (e.g. [81]). Second, the effects of recombination are more evident in *A. lyrata*: the median ρ/θ estimate in Germany is nearly 20 times that estimated for *A. thaliana* [79], and LD in Germany decays within hundreds of bases rather than thousands of bases in *A. thaliana* [83], [84]. Finally, the site frequency spectrum differs between the two species. Tajima's D is consistently positive within populations of *A. lyrata* (Figure 3), whereas world-wide samples of *A*. *thaliana* have a strongly negative D [79], [80].

Many of these differences between species can be attributed to differences in breeding system. While *A. thaliana* is almost exclusively inbreeding [85], *A lyrata* is a predominantly outcrossing species, though some degree of self-compatibility has been found in some ssp. lyrata pops from the Great Lakes region in the US and Canada [65]. Population genetic theory predicts, for example, lower diversity in selfing species both within populations [34], [86], and often species-wide [87], [88]. The observed difference in effective recombination rate is also approximately that expected due to differences in inbreeding. The ρ/θ ratio predicted in a selfing species is 1/(1-*F*) times smaller than in an equivalent outcrosser, where *F* is the inbreeding coefficient [89]. Comparing estimates of ρ/θ in *A. thaliana* and *A. lyrata* yields an estimated *F* of 0.95, and a selfing rate of 2*F*/(1+*F*) = 0.97 for *A. thaliana*, similar to selfing rate estimates from natural *A. thaliana* populations [85].

Differences in the site frequency spectrum as measured by Tajima's D, however, are not easily explained by differences in mating system. Estimates of Tajima's D can be strongly affected by sampling [13], [19], and virtually all *A*. *thaliana* data come from aggregated samples of a few plants from each of multiple populations [4], [79]–[81], [83]. To explore the effect of sampling differences between the two species, we generated 10,000 pseudo-random samples of two individuals from each of our six *A. lyrata* populations, thereby mimicking the *A. thaliana* sampling strategy of Nordborg *et al.* [79]. For each sample, we calculated D and the average θ_π_ per site for all sites (nonsynonymous and silent), and recorded the proportion of samples that produced values lower than those reported for *A. thaliana*. None of the *A. lyrata* pseudo-random samples yielded D or θ_π_ values approaching the *A. thaliana* data (D: −0.156–0.183 in *A. lyrata* samples *vs.* −0.793 for *A. thaliana*; θ_π_: 0.0093–0.0104 in *A. lyrata vs.* 0.0054 for *A. thaliana* [79], [80] ), suggesting that the difference in sampling alone is unlikely to explain discrepancies in the site frequency spectrum observed between the two species.

Demographic history may help to explain differences in the site frequency spectrum between species. Like *A. lyrata*, *A. thaliana* is hypothesized to have re-colonized Northern Europe after the last Ice Age [80], [90], and both species probably experienced periods of population bottlenecks followed by expansion. Unlike *A. lyrata*, however, *A. thaliana* is a weedy invasive, and the observed excess of rare variants in *A. thaliana* may be explained to some degree by metapopulation dynamics and continuing population expansion [91], [92]. In contrast, our demographic model suggests that population bottlenecks have had a lasting effect on diversity in *A. lyrata*, with many populations showing evidence of only a partial recovery. In this context it is interesting to note that, in spite of population genetic predictions of stronger population structure in selfing species [35], estimates of genetic structure in *A. thaliana* [79], [93] appear superficially similar to those reported here (Figure 5).

Life history and demographic processes may explain much of the difference between *A. thaliana* and *A. lyrata*, but selection may also play a significant role in shaping diversity. Purifying selection affects variation in both *A. thaliana* and *A. lyrata* [38], and we reasoned that similar evolutionary pressures might lead to correlations in diversity among loci. Comparing diversity in the loci sampled here to single feature polymorphism in their *A. thaliana* orthologues [94], however, we find little evidence for such correlations (Spearman's ρ = 0.20, p = 0.12 for a partial correlation correcting for divergence). Even if purifying selection plays a similar role in both species, there is reason to suspect that adaptive evolution might not. Although there have been few species-wide analyses in *A. lyrata*, evidence of adaptation in *A lyrata* to date has come from local populations, while *A*. *thaliana* seems to have experienced several selective sweeps across large parts of the species range [4], [95]. If cross-population sweeps are common in *A. thaliana*, selection – especially in conjunction with increased LD in a selfing species – may contribute substantially to the observed excess of rare mutations seen in species-wide samples of *A thaliana*.

In summary, we have presented the first large, multi-locus, multi-population survey of nucleotide diversity for *A. lyrata.* Our results underscore the importance of population-specific, non-equilibrium demographic processes in patterning diversity within *A. lyrata* and lay the groundwork for future studies of demographic history and local adaptation. We also highlight differences in patterns of polymorphism between *A. lyrata* and *A. thaliana* and discuss a host of factors that could contribute to these differences. Continued large-scale comparisons of diversity and divergence between *A. lyrata* and *A. thaliana* at both the population and species level will yield interesting insights into the forces that govern plant genome evolution.

## Supporting Information

Text S1 Command lines for coalescent simulation
(0.03 MB DOC)

Table S1 Loci studied. The number of silent sites, sample size in each population, and gene ontology terms are listed for the 77 loci studied.
(0.04 MB PDF)

Table S2 Diversity statistics. The number of segregating silent sites S, the number of silent singletons η_1_, the number of haplotypes N_h_, haplotype diversity H_e_, Watterson's estimate of diversity θ_w_, nucleotide diversity θ_π_, Tajima's D statistic, and the estimate of the recombination rate ρ are listed for each locus in each population.
(0.07 MB PDF)

Figure S1 Decline in linkage disequilibrium over distance. Plotted is a lowess regression fit of intralocus r^2^ against distance for all SNPs in all loci.
(0.59 MB TIF)
