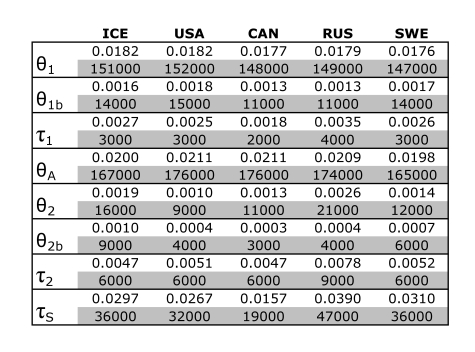# Correction: Patterns of Polymorphism and Demographic History in Natural Populations of *Arabidopsis lyrata*

**DOI:** 10.1371/annotation/83559ebe-03be-4bf7-a1b8-b34b2903742a

**Published:** 2010-08-18

**Authors:** Jeffrey Ross-Ibarra, Stephen I. Wright, John Paul Foxe, Akira Kawabe, Leah DeRose-Wilson, Gesseca Gos, Deborah Charlesworth, Brandon S. Gaut

In table 1, reported values of Ne were incorrectly converted from theta by a factor of 2. Values of divergence time are not affected. Please see the corrected table 1 here: 

**Figure:**