# Supplementary material for: Patterns of Polymorphism and Demographic History in Natural Populations of Arabidopsis lyrata
Source: PLoS One. 2008 Jun 11;3(6):e2411. doi: 10.1371/journal.pone.0002411 (PMC2408968; doi:10.1371/journal.pone.0002411)
Supplement: Text S1 — Command lines for coalescent simulation (0.03 MB DOC) [file pone.0002411.s001.doc]

## Supporting Text

The pairwise demographic model in Fig. 2A was generated with the following command line, utilizing the tbs feature of a modified version of Hudson’s ms software (Hudson 2002):

“msnsam tbs RUNS -t tbs -r tbs tbs -I 2 tbs tbs 0 -n 2 tbs -en tbs 2 tbs -en tbs 1 tbs -ej tbs 2 1 -eN tbs tbs -seed R1 R2 R3“

where RUNS = the product of the number of simulations and number of loci, R1-R3 are three random numbers, and msnsam is a modified version of ms that accepts the number of samples as an additional tbs argument. The command line used to simulate under the six population demographic model (Fig. 5b) was:

“msnsam tbs 10000 -t tbs -r tbs tbs -I 6 tbs tbs tbs tbs tbs tbs -n 2 tbs -n 3 tbs -n 4 tbs -n 5 tbs -n 6 tbs -en tbs 1 tbs -en tbs 2 tbs -en tbs 3 tbs -en tbs 4 tbs -en tbs 5 tbs -en tbs 6 tbs -ej tbs 2 1 -ej tbs 3 1 -ej tbs 4 1 -ej tbs 5 1 -ej tbs 6 1 -eN tbs tbs -seed R1 R2 R3”
