# Supplementary material for: Patterns of Polymorphism and Demographic History in Natural Populations of Arabidopsis lyrata
Source: PLoS One. 2008 Jun 11;3(6):e2411. doi: 10.1371/journal.pone.0002411 (PMC2408968; doi:10.1371/journal.pone.0002411)
Supplement: Table S1 — Loci studied. The number of silent sites, sample size in each population, and gene ontology terms are listed for the 77 loci studied. (0.04 MB PDF) [file pone.0002411.s002.pdf]

Table S1.

locus	Silent Sites	GER	CAN	USA	RUS	SWE	ICE	GO Terms
**AT1G72390**	74	20	20	22	26	16	20	expressed protein
**AT2G26140**	95	22	18	8	30	10	12	FtsH protease, putative
**AT3G44530**	118	18	18	18	28	16	18	transducin family protein / WD-40 repeat family
**AT4G16280**	271	10	10	10	10	10	14	flowering time control protein / FCA gamma
**AT4G25540**	357	10	10	10	10	10	14	DNA mismatch repair protein MSH3 (MSH3)
**AT4G38630**	269	10	10	10	10	10	14	26S proteasome regulatory subunit S5A (RPN10)
**AT2G44990**	100	12	4	8	30	16	14	armadillo/beta-catenin repeat family protein
**AT1G11050**	108	22	22	20	24	14	18	protein kinase family protein
**AT1G04650**	105	18	16	12	28	18	20	hypothetical protein
**AT1G06530**	81	22	24	10			22	myosin heavy chain-related
**AT4G36060**	247	10	10	10	10	10	14	basic helix-loop-helix (bHLH) family protein
**AT4G38160**	87	24	24	20	30	18	22	mitochondrial transcription termination
**AT3G13290**	110	14	14	16	30	18	8	transducin family protein / WD-40 repeat family
**AT3G51570**	79	12	18	10	22	18	18	disease resistance protein (TIR-NBS-LRR class)
**AT4G20410**	495	10	10	10	10	10	14	gamma-soluble NSF attachment protein
**AT2G41360**	99	22	20	14			16	F-box family protein
**AT3G23590**	130	24	22	18	26	18	22	expressed protein
**AT4G14180**	107	24	24	20	26	16	22	expressed protein
**AT4G18040**	329	10	10	10	10	10	14	eukaryotic translation initiation factor 4E 1
**AT2G26730**	81	18	14	12			14	leucine-rich repeat transmembrane protein
**AT2G47430**	78	20	18	20			14	cytokinin-responsive histidine kinase (CKI1)
**AT4G18520**	93	16	22	18	6	4	18	expressed protein
**AT5G04190**	109	22	22	22	28	8	8	phytochrome kinase substrate-related
**AT1G23200**	98	12	22	10	26	12	6	pectinesterase family protein
**AT4G40080**	223	10	10	10	10	10	14	epsin N-terminal homology (ENTH)
**AT5G20280**	112	22	22	18	24	12	22	sucrose-phosphate synthase, putative
**AT2G25050**	99	24	22	20	28	14	14	formin homology 2 domain-containing protein
**AT4G33250**	530	10	10	10	10	10	14	eukaryotic translation initiation factor 3
**AT4G28395**	380	10	10	10	10	10	14	lipid transfer protein, putative
**AT1G06520**	98	12	20	18	28	12	16	phospholipid/glycerol acyltransferase family
**AT3G55060**	90	22	24	14	30	16	22	expressed protein
**AT1G31930**	90	16	18	16	22	12	20	extra-large guanine nucleotide binding protein
**AT1G78850**	110	24	24	18	28	18	18	curculin-like (mannose-binding) lectin family
**AT4G00030**	245	8	10	10	10	10	14	plastid-lipid associated protein PAP
**AT3G50740**	103	22	22	20	28	14	20	UDP-glucoronosyl / UDP-glucosyl transferase family
**AT3G10340**	103	22	20	18	26	18	18	phenylalanine ammonia-lyase, putative
**AT4G08170**	412	10	10	10	10	10	14	inositol 1,3,4-trisphosphate 5/6-kinase family
**AT1G68520**	93	20		4	20	16	20	zinc finger (B-box type) family protein
**AT4G39680**	103	16	22	20			14	SAP domain-containing protein
**AT1G15240**	86	20	20	16	30	18	20	phox (PX) domain-containing protein
**AT1G64170**	103	20	24	12	30	18	8	cation/hydrogen exchanger, putative (CHX16)
**AT3G54720**	136	22	24	22	26	12	22	glutamate carboxypeptidase, putative (AMP1)
**AT2G36980**	101	8	20	20	30	18	22	pentatricopeptide (PPR) repeat-containing
**AT3G62890**	94	20	22	16	26	16	16	pentatricopeptide (PPR) repeat-containing

locus	Silent Sites	GER	CAN	USA	RUS	SWE	ICE	GO Terms
**AT4G02390**	287	10	10	10	10	10	14	poly (ADP-ribose) polymerase / NAD(+)
**AT5G53020**	103	14	8	8	30	8	10	expressed protein
**AT1G65450**	100	20	24	18	30	14	20	transferase family protein
**AT1G68530**	78	16	22	18	30	18	18	very-long-chain fatty acid condensing enzyme
**AT4G04350**	313	10	10	10	10	10	14	leucyl-tRNA synthetase, putative / leucine--tRNA
**AT2G43680**	114	10	12	6	26	14		calmodulin-binding family protein
**AT1G10900**	100	24	22	16	24	14	18	phosphatidylinositol-4-phosphate 5-kinase family
**AT1G62310**	88	16	22	18	26	16	18	transcription factor jumonji (jmjC)
**AT1G62520**	92	14	24	18	30	12	14	expressed protein
**AT4G10340**	386	10	10	10	10	10	14	chlorophyll A-B binding protein CP26
**AT5G48100**	109	24	24	22	30	18	22	laccase family protein / diphenol oxidase family
**AT1G62390**	97	18	20	18	24	10	20	octicosapeptide/Phox/Bem1p (PB1)
**AT4G22720**	271	10	10	10	10	10	14	glycoprotease M22 family protein
**AT5G66280**	99	20	22	22	30	16	18	GDP-D-mannose 4,6-dehydratase, putative
**AT2G46550**	95	20	18	6	30	16	16	expressed protein
**AT1G10980**	101	16	18	14	28	18	14	expressed protein
**AT4G08840**	89	4	20	18	20	18	8	pumilio/Puf RNA-binding domain-containing
**AT4G14210**	413	10	10	10	10	10	14	phytoene dehydrogenase, chloroplast
**AT5G43670**	108	16	10	16			22	transport protein, putative
**AT1G01040**	90	18	22	16	26	14	16	DEAD/DEAH box helicase carpel factory
**AT3G20820**	114	18	6	4	8	6	16	leucine-rich repeat family protein
**AT5G51670**	120	20	18	22			12	expressed protein
**AT4G12030**	279	10	10	10	10	10	14	bile acid:sodium symporter family protein
**AT4G30950**	331	10	10	10	10	10	14	omega-6 fatty acid desaturase, chloroplast
**AT2G16870**	111	18	18	18	4	10	20	disease resistance protein (TIR-NBS-LRR class)
**AT1G03560**	98	22	18	20	30	16	16	pentatricopeptide (PPR) repeat-containing
**AT5G41920**	108	16	16	10			6	scarecrow transcription factor family protein
**AT3G48690**	96	24	22	12	24	14	18	expressed protein
**AT2G23170**	97	22	22	8	30	16	16	auxin-responsive GH3 family protein
**AT2G28050**	90	24	20	18	26	16	22	pentatricopeptide (PPR) repeat-containing
**AT4G14190**	102	16	16	18	26	16	18	pentatricopeptide (PPR) repeat-containing
**AT1G59720**	99	14	12	16	16	10	14	pentatricopeptide (PPR) repeat-containing
**AT1G74600**	107	22	24	16	14	16	20	pentatricopeptide (PPR) repeat-containing
