# Supplementary material for: Patterns of Polymorphism and Demographic History in Natural Populations of Arabidopsis lyrata
Source: PLoS One. 2008 Jun 11;3(6):e2411. doi: 10.1371/journal.pone.0002411 (PMC2408968; doi:10.1371/journal.pone.0002411)
Supplement: Table S2 — Diversity statistics. The number of segregating silent sites S, the number of silent singletons η1, the number of haplotypes Nh, haplotype diversity He, Watterson's estimate of diversity θw, nucleotide diversity θπ, Tajima's D statistic, and the estimate of the recombination rate ρ are listed for each locus in each population. (0.07 MB PDF) [file pone.0002411.s003.pdf]

Table S2.

locus	pop.	S	η_1	N_h	H_e	θ_w	θ_π	D	ρ
AT1G01040	can	2	0	2	0.485	0.549	0.970	1.739	
AT1G03560	can	1	1	2	0.111	0.291	0.111	-1.165	
AT1G04650	can	0	0	1	0.000	0.000	0.000		
AT1G06520	can	1	0	2	0.268	0.282	0.268	-0.086	
AT1G06530	can	2	0	3	0.649	0.536	0.808	1.118	
AT1G10900	can	0	0	1	0.000	0.000	0.000		
AT1G10980	can	0	0	1	0.000	0.000	0.000		
AT1G11050	can	0	0	1	0.000	0.000	0.000		
AT1G15240	can	0	0	1	0.000	0.000	0.000		
AT1G23200	can	0	0	1	0.000	0.000	0.000		
AT1G31930	can	1	1	2	0.111	0.291	0.111	-1.165	
AT1G59720	can	17	1	5	0.742	5.629	6.606	0.753	0
AT1G62310	can	0	0	1	0.000	0.000	0.000		
AT1G62390	can	1	1	2	0.100	0.282	0.100	-1.164	
AT1G62520	can	0	0	1	0.000	0.000	0.000		
AT1G64170	can	4	0	3	0.594	1.071	1.377	0.780	0
AT1G65450	can	5	0	4	0.665	1.352	1.680	0.709	45
AT1G68530	can	1	1	2	0.091	0.274	0.091	-1.162	
AT1G72390	can	0	0	1	0.000	0.000	0.000		
AT1G74600	can	0	0	1	0.000	0.000	0.000		
AT1G78850	can	3	0	5	0.819	0.803	1.293	1.535	0
AT2G16870	can	1	0	2	0.366	0.291	0.366	0.488	
AT2G23170	can	2	0	3	0.567	0.549	1.043	2.043	
AT2G25050	can	0	0	1	0.000	0.000	0.000		
AT2G26140	can	0	0	1	0.000	0.000	0.000		
AT2G26730	can	3	0	4	0.582	0.943	0.890	-0.173	0
AT2G28050	can	0	0	1	0.000	0.000	0.000		
AT2G36980	can	0	0	1	0.000	0.000	0.000		
AT2G41360	can	1	0	2	0.337	0.282	0.337	0.352	
AT2G43680	can	1	0	2	0.545	0.331	0.545	1.486	
AT2G44990	can	0	0	1	0.000	0.000	0.000		
AT2G46550	can	0	0	1	0.000	0.000	0.000		
AT2G47430	can	2	0	4	0.647	0.581	0.928	1.455	
AT3G10340	can	1	0	2	0.189	0.282	0.189	-0.592	
AT3G13290	can	0	0	1	0.000	0.000	0.000		
AT3G20820	can	0	0	1	0.000	0.000	0.000		
AT3G23590	can	0	0	1	0.000	0.000	0.000		
AT3G44530	can	1	0	2	0.366	0.291	0.366	0.488	
AT3G48690	can	2	0	2	0.312	0.549	0.623	0.309	
AT3G50740	can	5	0	8	0.887	1.372	2.229	1.859	0
AT3G51570	can	0	0	1	0.000	0.000	0.000		
AT3G54720	can	2	0	4	0.750	0.536	0.982	1.832	
AT3G55060	can	0	0	1	0.000	0.000	0.000		
AT3G62890	can	1	0	2	0.368	0.274	0.368	0.593	
AT4G00030	can	0	0	1	0.000	0.000	0.000		
AT4G02390	can	1	0	2	0.533	0.353	0.533	1.303	

locus	pop.	S	η_1	N_h	H_e	θ_w	θ_π	D	ρ
AT4G04350	can	0	0	1	0.000	0.000	0.000		
AT4G08170	can	0	0	1	0.000	0.000	0.000		
AT4G08840	can	1	0	2	0.521	0.282	0.521	1.531	
AT4G10340	can	7	1	4	0.644	2.474	2.333	-0.241	0
AT4G12030	can	33	4	6	0.889	11.665	15.200	1.461	0
AT4G14180	can	2	2	3	0.163	0.536	0.167	-1.515	
AT4G14190	can	17	17	3	0.242	5.123	2.125	-2.321	0
AT4G14210	can	1	0	2	0.356	0.353	0.356	0.015	
AT4G16280	can	0	0	1	0.000	0.000	0.000		
AT4G18040	can	4	2	3	0.644	1.414	1.467	0.143	0
AT4G18520	can	1	0	2	0.485	0.274	0.485	1.334	
AT4G20410	can	0	0	1	0.000	0.000	0.000		
AT4G22720	can	0	0	1	0.000	0.000	0.000		
AT4G25540	can	0	0	1	0.000	0.000	0.000		
AT4G28395	can	0	0	1	0.000	0.000	0.000		
AT4G30950	can	5	0	2	0.467	1.767	2.333	1.284	0
AT4G33250	can	4	2	4	0.367	1.498	1.489	-0.026	0
AT4G36060	can	0	0	1	0.000	0.000	0.000		
AT4G38160	can	0	0	1	0.000	0.000	0.000		
AT4G38630	can	2	0	2	0.356	0.707	0.711	0.019	
AT4G39680	can	0	0	1	0.000	0.000	0.000		
AT4G40080	can	1	0	2	0.356	0.353	0.356	0.015	
AT5G04190	can	2	1	3	0.541	0.549	0.576	0.112	
AT5G20280	can	0	0	1	0.000	0.000	0.000		
AT5G41920	can	0	0	1	0.000	0.000	0.000		
AT5G43670	can	9	1	6	0.844	3.181	3.267	0.117	0
AT5G48100	can	9	0	6	0.851	2.410	3.431	1.389	0
AT5G51670	can	0	0	1	0.000	0.000	0.000		
AT5G53020	can	1	1	2	0.250	0.386	0.250	-1.055	
AT5G66280	can	1	0	2	0.416	0.274	0.416	0.895	
AT1G01040	ger	6	0	4	0.791	1.744	3.176	2.703	0
AT1G03560	ger	16	1	6	0.632	4.389	5.416	0.849	0
AT1G04650	ger	2	2	3	0.216	0.581	0.222	-1.508	
AT1G06520	ger	3	2	4	0.636	0.993	0.742	-0.829	
AT1G06530	ger	1	0	2	0.173	0.274	0.173	-0.641	
AT1G10900	ger	7	0	9	0.870	1.875	2.424	0.916	3
AT1G10980	ger	10	2	12	0.967	3.014	3.367	0.437	9
AT1G11050	ger	2	2	3	0.177	0.549	0.182	-1.515	
AT1G15240	ger	4	1	4	0.642	1.127	1.205	0.200	
AT1G23200	ger	2	0	3	0.545	0.662	0.606	-0.248	
AT1G31930	ger	3	1	4	0.692	0.904	0.975	0.227	
AT1G59720	ger	28	8	12	0.967	8.805	9.341	0.260	3
AT1G62310	ger	9	4	7	0.792	2.712	2.183	-0.716	0
AT1G62390	ger	6	1	5	0.758	1.744	2.627	1.667	0
AT1G62520	ger	6	0	7	0.912	1.887	2.385	0.947	0
AT1G64170	ger	8	3	5	0.626	2.255	1.474	-1.168	0
AT1G65450	ger	7	2	10	0.911	1.973	1.916	-0.095	92
AT1G68520	ger	7	1	4	0.437	1.973	1.237	-1.227	0

locus	pop.	S	η_1	N_h	H_e	θ_w	θ_π	D	ρ
AT1G68530	ger	4	1	5	0.817	1.205	1.675	1.219	
AT1G72390	ger	0	0	1	0.000	0.000	0.000		
AT1G74600	ger	21	0	5	0.792	5.761	10.584	3.126	0
AT1G78850	ger	3	0	4	0.764	0.803	1.210	1.274	
AT2G16870	ger	13	3	13	0.967	3.780	5.052	1.256	23
AT2G23170	ger	15	0	14	0.965	4.115	5.775	1.455	4
AT2G25050	ger	2	0	3	0.518	0.536	0.692	0.642	
AT2G26140	ger	0	0	1	0.000	0.000	0.000		
AT2G26730	ger	4	4	3	0.216	1.163	0.444	-1.853	
AT2G28050	ger	18	2	22	0.993	4.820	6.547	1.292	22
AT2G36980	ger	4	1	6	0.929	1.543	1.643	0.283	
AT2G41360	ger	1	0	2	0.416	0.274	0.416	0.895	
AT2G43680	ger	8	4	4	0.778	2.828	2.578	-0.382	1
AT2G44990	ger	1	1	2	0.167	0.331	0.167	-1.141	
AT2G46550	ger	7	0	7	0.863	1.973	2.963	1.650	0
AT2G47430	ger	2	1	3	0.416	0.564	0.437	-0.528	
AT3G10340	ger	5	2	7	0.792	1.372	1.225	-0.317	100
AT3G13290	ger	2	1	3	0.385	0.629	0.407	-0.959	
AT3G20820	ger	10	0	12	0.935	2.907	4.124	1.507	47
AT3G23590	ger	2	0	3	0.507	0.536	0.551	0.062	
AT3G44530	ger	0	0	1	0.000	0.000	0.000		
AT3G48690	ger	13	2	15	0.946	3.481	5.442	1.954	12
AT3G50740	ger	4	1	7	0.766	1.097	1.199	0.261	
AT3G51570	ger	1	0	2	0.303	0.331	0.303	-0.195	
AT3G54720	ger	5	0	8	0.896	1.372	2.117	1.615	0
AT3G55060	ger	5	3	6	0.667	1.372	0.900	-1.021	0
AT3G62890	ger	6	0	4	0.500	1.691	1.532	-0.301	4
AT4G00030	ger	6	1	7	0.964	2.314	2.750	0.878	4
AT4G02390	ger	14	2	4	0.533	4.949	4.778	-0.158	4
AT4G04350	ger	18	2	5	0.756	6.427	6.898	0.345	0
AT4G08170	ger	12	4	7	0.933	4.242	5.111	0.926	5
AT4G08840	ger	5	2	3	0.833	2.727	3.000	0.956	3
AT4G10340	ger	26	7	10	1.000	9.191	10.067	0.455	41
AT4G12030	ger	29	7	8	0.933	10.251	11.178	0.433	2
AT4G14180	ger	1	0	2	0.464	0.268	0.464	1.232	
AT4G14190	ger	20	1	10	0.933	6.027	7.658	1.089	3
AT4G14210	ger	31	9	7	0.911	11.534	14.189	1.165	3
AT4G16280	ger	0	0	1	0.000	0.000	0.000		
AT4G18040	ger	3	0	3	0.733	1.060	1.533	1.604	
AT4G18520	ger	1	0	2	0.525	0.301	0.525	1.474	
AT4G20410	ger	7	4	5	0.756	2.474	1.978	-0.850	13
AT4G22720	ger	17	0	6	0.911	6.201	7.867	1.289	13
AT4G25540	ger	1	1	2	0.200	0.353	0.200	-1.112	
AT4G28395	ger	11	9	5	0.100	3.985	2.795	-1.370	11
AT4G30950	ger	32	3	8	0.956	11.312	13.933	1.116	11
AT4G33250	ger	11	7	7	0.633	4.599	3.745	-0.983	9
AT4G36060	ger	2	1	3	0.600	0.707	0.667	-0.184	
AT4G38160	ger	2	1	3	0.236	0.536	0.243	-1.202	

locus	pop.	S	η_1	N_h	H_e	θ_w	θ_π	D	ρ
AT4G38630	ger	1	0	2	0.356	0.353	0.356	0.015	
AT4G39680	ger	3	0	3	0.658	0.904	1.392	1.563	
AT4G40080	ger	4	1	4	0.733	1.498	1.444	-0.147	
AT5G04190	ger	3	1	5	0.468	0.823	0.649	-0.546	
AT5G20280	ger	4	3	4	0.571	1.097	0.779	-0.814	
AT5G41920	ger	13	0	7	0.867	3.918	6.050	2.099	0
AT5G43670	ger	13	4	10	0.925	3.918	3.733	-0.182	10
AT5G48100	ger	9	1	8	0.826	2.410	2.920	0.694	4
AT5G51670	ger	13	1	14	0.968	3.664	4.747	1.072	9
AT5G53020	ger	11	8	7	0.758	3.459	1.934	-1.738	2
AT5G66280	ger	6	0	5	0.758	1.691	2.926	2.327	0
AT1G01040	ice	2	0	3	0.633	0.603	0.767	0.696	
AT1G03560	ice	15	0	4	0.600	4.520	3.667	-0.740	0
AT1G04650	ice	0	0	1	0.000	0.000	0.000		
AT1G06520	ice	0	0	1	0.000	0.000	0.000		
AT1G06530	ice	1	0	2	0.485	0.274	0.485	1.334	
AT1G10900	ice	5	0	4	0.542	1.454	1.301	-0.333	2
AT1G10980	ice	2	2	2	0.143	0.629	0.286	-1.481	
AT1G11050	ice	1	1	2	0.111	0.291	0.111	-1.165	
AT1G15240	ice	0	0	1	0.000	0.000	0.000		
AT1G23200	ice	2	0	2	0.533	0.876	1.067	1.032	
AT1G31930	ice	3	0	3	0.626	0.846	1.068	0.704	0
AT1G59720	ice	21	5	8	0.934	6.604	7.890	0.817	4
AT1G62310	ice	1	0	2	0.294	0.291	0.294	0.022	
AT1G62390	ice	6	5	4	0.521	1.700	1.029	-1.264	0
AT1G62520	ice	2	0	3	0.659	0.629	0.791	0.700	
AT1G64170	ice	2	0	2	0.536	0.771	1.071	1.449	
AT1G65450	ice	5	0	6	0.832	1.409	2.053	1.397	0
AT1G68520	ice	0	0	1	0.000	0.000	0.000		
AT1G68530	ice	0	0	1	0.000	0.000	0.000		
AT1G72390	ice	0	0	1	0.000	0.000	0.000		
AT1G74600	ice	1	0	2	0.505	0.282	0.505	1.430	
AT1G78850	ice	2	0	4	0.765	0.581	1.007	1.784	
AT2G16870	ice	9	0	4	0.574	2.537	4.311	2.404	0
AT2G23170	ice	11	0	3	0.592	3.315	5.858	2.899	0
AT2G25050	ice	1	0	2	0.495	0.314	0.495	1.212	
AT2G26140	ice	0	0	1	0.000	0.000	0.000		
AT2G26730	ice	6	3	7	0.802	1.887	1.758	-0.244	6
AT2G28050	ice	5	1	4	0.697	1.372	1.753	0.827	0
AT2G36980	ice	5	0	4	0.662	1.372	1.814	0.958	2
AT2G41360	ice	0	0	1	0.000	0.000	0.000		
AT2G44990	ice	0	0	1	0.000	0.000	0.000		
AT2G46550	ice	1	0	2	0.458	0.301	0.458	1.034	
AT2G47430	ice	2	0	2	0.538	0.629	1.077	1.933	
AT3G10340	ice	1	0	2	0.529	0.291	0.529	1.548	
AT3G13290	ice	2	1	3	0.607	0.771	0.679	-0.448	
AT3G20820	ice	2	0	4	0.692	0.603	0.850	1.050	
AT3G23590	ice	2	0	2	0.312	0.549	0.623	0.309	

locus	pop.	S	η_1	N_h	H_e	θ_w	θ_π	D	ρ
AT3G44530	ice	1	1	2	0.111	0.291	0.111	-1.165	
AT3G48690	ice	15	2	8	0.856	4.361	6.373	1.749	0
AT3G50740	ice	9	0	7	0.737	2.537	3.063	0.713	0
AT3G51570	ice	4	0	5	0.791	1.163	1.895	1.889	0
AT3G54720	ice	2	0	2	0.312	0.549	0.623	0.309	
AT3G55060	ice	2	0	4	0.693	0.549	0.900	1.453	
AT3G62890	ice	9	0	11	0.950	2.725	4.445	2.328	10
AT4G00030	ice	8	2	3	0.538	2.516	3.253	1.105	0
AT4G02390	ice	5	0	3	0.604	1.572	2.681	2.442	0
AT4G04350	ice	2	1	3	0.385	0.629	0.505	-0.532	
AT4G08170	ice	5	0	2	0.527	1.572	2.637	2.345	0
AT4G08840	ice	0	0	1	0.000	0.000	0.000		
AT4G10340	ice	15	0	6	0.868	4.717	6.407	1.461	0
AT4G12030	ice	37	1	6	0.747	11.635	14.912	1.221	0
AT4G14180	ice	1	0	2	0.247	0.274	0.247	-0.175	
AT4G14190	ice	4	0	2	0.471	1.163	1.882	1.855	0
AT4G14210	ice	6	0	3	0.615	1.887	2.374	0.926	0
AT4G16280	ice	0	0	1	0.000	0.000	0.000		
AT4G18040	ice	7	0	4	0.648	2.201	3.297	1.837	0
AT4G18520	ice	1	0	2	0.209	0.291	0.209	-0.529	
AT4G20410	ice	10	1	9	0.654	3.216	4.063	1.049	6
AT4G22720	ice	7	0	5	0.835	2.201	2.857	1.100	7
AT4G25540	ice	2	0	2	0.264	0.629	0.527	-0.438	
AT4G28395	ice	15	0	3	0.560	4.717	5.275	0.482	7
AT4G30950	ice	6	0	3	0.484	1.887	1.758	-0.244	0
AT4G33250	ice	1	0	2	0.440	0.314	0.440	0.842	
AT4G36060	ice	3	1	3	0.385	0.943	0.670	-0.886	0
AT4G38160	ice	0	0	1	0.000	0.000	0.000		
AT4G38630	ice	2	0	2	0.538	0.629	1.077	1.933	
AT4G39680	ice	0	0	1	0.000	0.000	0.000		
AT4G40080	ice	4	4	2	0.143	1.258	0.571	-1.798	0
AT5G04190	ice	5	0	2	0.429	1.928	2.143	0.504	0
AT5G20280	ice	3	0	6	0.797	0.823	1.143	1.005	0
AT5G41920	ice	4	0	2	0.533	1.752	2.133	1.181	0
AT5G43670	ice	10	0	13	0.952	2.743	4.316	1.955	0
AT5G48100	ice	7	2	3	0.255	1.920	1.342	-0.964	0
AT5G51670	ice	9	0	3	0.545	2.980	2.727	-0.345	0
AT5G53020	ice	6	0	3	0.733	2.121	2.800	1.325	0
AT5G66280	ice	6	0	2	0.209	1.744	1.255	-0.924	0
AT1G01040	rus	0	0	1	0.000	0.000	0.000		
AT1G03560	rus	15	1	5	0.455	3.786	4.090	0.270	0
AT1G04650	rus	0	0	1	0.000	0.000	0.000		
AT1G06520	rus	0	0	1	0.000	0.000	0.000		
AT1G10900	rus	0	0	1	0.000	0.000	0.000		
AT1G10980	rus	4	4	3	0.140	1.028	0.286	-1.889	0
AT1G11050	rus	8	7	4	0.543	2.142	1.014	-1.689	0
AT1G15240	rus	3	0	3	0.248	0.757	0.386	-1.153	0
AT1G23200	rus	6	0	7	0.818	1.572	2.526	1.796	0

locus	pop.	S	η_1	N_h	H_e	θ_w	θ_π	D	ρ
AT1G31930	rus	1	0	2	0.519	0.274	0.519	1.554	
AT1G59720	rus	11	11	3	0.242	3.315	1.375	-2.211	0
AT1G62310	rus	0	0	1	0.000	0.000	0.000		
AT1G62390	rus	1	0	2	0.518	0.268	0.518	1.573	
AT1G62520	rus	10	0	7	0.864	2.524	4.634	2.648	0
AT1G64170	rus	5	0	4	0.754	1.262	2.483	2.639	0
AT1G65450	rus	4	0	4	0.600	1.010	1.480	1.197	0
AT1G68520	rus	2	0	2	0.189	0.583	0.423	-0.667	
AT1G68530	rus	4	0	5	0.726	1.010	1.214	0.519	0
AT1G72390	rus	0	0	1	0.000	0.000	0.000		
AT1G74600	rus	0	0	1	0.000	0.000	0.000		
AT1G78850	rus	0	0	1	0.000	0.000	0.000		
AT2G16870	rus	1	0	2	0.667	0.545	0.667	1.633	
AT2G23170	rus	0	0	1	0.000	0.000	0.000		
AT2G25050	rus	0	0	1	0.000	0.000	0.000		
AT2G26140	rus	2	0	4	0.533	0.505	0.662	0.638	
AT2G28050	rus	0	0	1	0.000	0.000	0.000		
AT2G36980	rus	3	0	4	0.600	0.757	1.110	1.097	0
AT2G43680	rus	6	0	4	0.763	1.572	3.102	2.879	0
AT2G44990	rus	0	0	1	0.000	0.000	0.000		
AT2G46550	rus	1	0	2	0.515	0.252	0.515	1.621	
AT3G10340	rus	12	0	6	0.751	3.145	5.588	2.616	0
AT3G13290	rus	0	0	1	0.000	0.000	0.000		
AT3G20820	rus	2	1	3	0.607	0.771	0.679	-0.448	
AT3G23590	rus	0	0	1	0.000	0.000	0.000		
AT3G44530	rus	0	0	1	0.000	0.000	0.000		
AT3G48690	rus	6	6	3	0.163	1.607	0.500	-2.083	0
AT3G50740	rus	0	0	1	0.000	0.000	0.000		
AT3G51570	rus	0	0	1	0.000	0.000	0.000		
AT3G54720	rus	0	0	1	0.000	0.000	0.000		
AT3G55060	rus	2	1	3	0.421	0.505	0.437	-0.276	
AT3G62890	rus	8	0	7	0.806	2.096	3.194	1.646	0
AT4G00030	rus	6	0	2	0.533	2.121	3.200	2.105	0
AT4G02390	rus	15	2	5	0.400	5.387	5.200	-0.162	0
AT4G04350	rus	3	0	2	0.467	1.145	1.467	1.092	0
AT4G08170	rus	0	0	1	0.000	0.000	0.000		
AT4G08840	rus	0	0	1	0.000	0.000	0.000		
AT4G10340	rus	0	0	1	0.000	0.000	0.000		
AT4G12030	rus	4	4	2	0.200	1.414	0.800	-1.667	0
AT4G14180	rus	2	0	3	0.283	0.524	0.295	-0.935	
AT4G14190	rus	7	0	3	0.283	1.834	1.034	-1.336	0
AT4G14210	rus	2	1	2	0.356	0.791	0.689	-0.468	
AT4G16280	rus	2	1	3	0.511	0.707	0.667	-0.184	
AT4G18040	rus	1	1	2	0.200	0.353	0.200	-1.112	
AT4G18520	rus	0	0	1	0.000	0.000	0.000		
AT4G20410	rus	3	3	3	0.378	1.060	0.600	-1.562	0
AT4G22720	rus	0	0	1	0.000	0.000	0.000		
AT4G25540	rus	0	0	1	0.000	0.000	0.000		

locus	pop.	S	η_1	N_h	H_e	θ_w	θ_π	D	ρ
AT4G28395	rus	5	4	3	0.378	2.021	1.556	-1.056	0
AT4G30950	rus	1	0	1	0.000	0.438	0.600	1.174	
AT4G33250	rus	3	1	3	0.511	1.060	1.022	-0.130	0
AT4G36060	rus	0	0	1	0.000	0.000	0.000		
AT4G38160	rus	0	0	1	0.000	0.000	0.000		
AT4G38630	rus	0	0	1	0.000	0.000	0.000		
AT4G40080	rus	7	3	3	0.378	2.474	2.022	-0.774	0
AT5G04190	rus	1	0	2	0.508	0.257	0.508	1.557	
AT5G20280	rus	0	0	1	0.000	0.000	0.000		
AT5G48100	rus	4	0	3	0.683	1.010	1.922	2.320	0
AT5G53020	rus	0	0	1	0.000	0.000	0.000		
AT5G66280	rus	3	0	4	0.708	0.757	1.379	1.933	0
AT1G01040	swe	2	0	3	0.670	0.629	0.978	1.506	
AT1G03560	swe	15	0	3	0.567	4.520	3.667	-0.740	0
AT1G04650	swe	0	0	1	0.000	0.000	0.000		
AT1G06520	swe	2	0	4	0.803	0.662	1.061	1.758	
AT1G10900	swe	4	0	5	0.802	1.258	1.978	1.886	0
AT1G10980	swe	2	0	3	0.601	0.581	0.732	0.632	
AT1G11050	swe	0	0	1	0.000	0.000	0.000		
AT1G15240	swe	0	0	1	0.000	0.000	0.000		
AT1G23200	swe	3	0	5	0.788	0.993	1.227	0.772	0
AT1G31930	swe	0	0	1	0.000	0.000	0.000		
AT1G59720	swe	6	0	4	0.800	2.121	3.200	2.105	0
AT1G62310	swe	0	0	1	0.000	0.000	0.000		
AT1G62390	swe	6	5	4	0.644	2.121	1.356	-1.493	0
AT1G62520	swe	4	0	5	0.848	1.325	1.636	0.828	0
AT1G64170	swe	3	0	3	0.686	0.872	1.275	1.279	0
AT1G65450	swe	1	0	2	0.495	0.314	0.495	1.212	
AT1G68520	swe	0	0	1	0.000	0.000	0.000		
AT1G68530	swe	3	0	6	0.856	0.872	1.431	1.778	0
AT1G72390	swe	0	0	1	0.000	0.000	0.000		
AT1G74600	swe	1	0	2	0.325	0.301	0.325	0.156	
AT1G78850	swe	2	0	3	0.392	0.581	0.418	-0.685	
AT2G16870	swe	13	4	9	0.978	4.628	4.860	0.231	20
AT2G23170	swe	13	0	5	0.733	3.918	3.333	-0.575	0
AT2G25050	swe	1	0	2	0.440	0.314	0.440	0.842	
AT2G26140	swe	0	0	1	0.000	0.000	0.000		
AT2G28050	swe	8	4	8	0.875	2.411	2.467	0.083	0
AT2G36980	swe	3	0	3	0.464	0.872	0.797	-0.238	0
AT2G43680	swe	2	2	2	0.143	0.629	0.286	-1.481	
AT2G44990	swe	0	0	1	0.000	0.000	0.000		
AT2G46550	swe	3	0	4	0.775	0.904	1.175	0.869	0
AT3G10340	swe	1	0	2	0.425	0.291	0.425	0.870	
AT3G13290	swe	1	0	2	0.209	0.291	0.209	-0.529	
AT3G20820	swe	5	3	3	0.733	2.190	2.067	-0.315	0
AT3G23590	swe	2	0	2	0.294	0.581	0.588	0.028	
AT3G44530	swe	0	0	1	0.000	0.000	0.000		
AT3G48690	swe	1	0	2	0.363	0.314	0.363	0.324	

locus	pop.	S	η_1	N_h	H_e	θ_w	θ_π	D	ρ
AT3G50740	swe	3	0	4	0.758	0.943	1.484	1.753	0
AT3G51570	swe	3	0	4	0.680	0.872	1.307	1.383	0
AT3G54720	swe	0	0	1	0.000	0.000	0.000		
AT3G55060	swe	2	1	3	0.575	0.603	0.650	0.201	
AT3G62890	swe	6	6	3	0.242	1.808	0.750	-2.003	0
AT4G00030	swe	3	0	2	0.467	1.060	1.400	1.152	0
AT4G02390	swe	2	0	3	0.511	0.707	0.822	0.526	
AT4G04350	swe	1	0	2	0.222	0.386	0.429	0.311	
AT4G08170	swe	6	0	2	0.556	2.121	3.333	2.365	0
AT4G08840	swe	0	0	1	0.000	0.000	0.000		
AT4G10340	swe	3	0	2	0.356	1.060	1.067	0.021	0
AT4G12030	swe	0	0	1	0.000	0.000	0.000		
AT4G14180	swe	2	0	4	0.725	0.603	0.925	1.369	
AT4G14190	swe	18	0	7	0.892	5.425	8.308	2.119	0
AT4G14210	swe	0	0	1	0.000	0.000	0.000		
AT4G16280	swe	3	0	4	0.822	1.060	1.356	1.001	0
AT4G18040	swe	2	0	3	0.511	0.707	0.822	0.526	
AT4G18520	swe	0	0	1	0.000	0.000	0.000		
AT4G20410	swe	5	2	3	0.600	1.767	1.867	0.225	3
AT4G22720	swe	7	2	5	0.844	2.474	2.356	-0.203	3
AT4G25540	swe	1	0	2	0.356	0.353	0.356	0.015	
AT4G28395	swe	14	11	6	0.744	5.310	4.233	-0.996	4
AT4G30950	swe	1	0	2	0.467	0.353	0.467	0.820	
AT4G33250	swe	0	0	1	0.000	0.000	0.000		
AT4G36060	swe	1	0	2	0.356	0.353	0.356	0.015	
AT4G38160	swe	0	0	1	0.000	0.000	0.000		
AT4G38630	swe	0	0	1	0.000	0.000	0.000		
AT4G40080	swe	6	0	4	-0.033	2.205	2.422	0.423	0
AT5G04190	swe	8	0	4	0.821	3.085	3.536	0.706	0
AT5G20280	swe	3	0	6	0.879	0.993	1.591	1.973	0
AT5G48100	swe	6	0	5	0.556	1.744	1.595	-0.282	2
AT5G53020	swe	7	0	2	0.571	2.700	4.000	2.292	0
AT5G66280	swe	0	0	1	0.000	0.000	0.000		
AT1G01040	usa	1	0	2	0.233	0.301	0.233	-0.448	
AT1G03560	usa	1	0	2	0.189	0.282	0.189	-0.592	
AT1G04650	usa	0	0	1	0.000	0.000	0.000		
AT1G06520	usa	1	1	2	0.111	0.291	0.111	-1.165	
AT1G06530	usa	1	0	2	0.356	0.353	0.356	0.015	
AT1G10900	usa	6	0	5	0.742	1.808	3.150	2.540	0
AT1G10980	usa	0	0	1	0.000	0.000	0.000		
AT1G11050	usa	0	0	1	0.000	0.000	0.000		
AT1G15240	usa	0	0	1	0.000	0.000	0.000		
AT1G23200	usa	2	0	4	0.822	0.707	1.111	1.844	
AT1G31930	usa	1	1	2	0.125	0.301	0.125	-1.162	
AT1G59720	usa	4	0	3	0.433	1.205	0.933	-0.706	0
AT1G62310	usa	0	0	1	0.000	0.000	0.000		
AT1G62390	usa	1	0	2	0.425	0.291	0.425	0.870	
AT1G62520	usa	5	0	4	0.739	1.454	2.516	2.314	0

locus	pop.	S	η_1	N_h	H_e	θ_w	θ_π	D	ρ
AT1G64170	usa	0	0	1	0.000	0.000	0.000		
AT1G65450	usa	6	0	9	0.902	1.744	2.739	1.876	10
AT1G68520	usa	0	0	1	0.000	0.000	0.000		
AT1G68530	usa	0	0	1	0.000	0.000	0.000		
AT1G72390	usa	0	0	1	0.000	0.000	0.000		
AT1G74600	usa	0	0	1	0.000	0.000	0.000		
AT1G78850	usa	0	0	1	0.000	0.000	0.000		
AT2G16870	usa	0	0	1	0.000	0.000	0.000		
AT2G23170	usa	0	0	1	0.000	0.000	0.000		
AT2G25050	usa	0	0	1	0.000	0.000	0.000		
AT2G26140	usa	0	0	1	0.000	0.000	0.000		
AT2G26730	usa	4	0	3	0.682	1.325	1.636	0.828	0
AT2G28050	usa	0	0	1	0.000	0.000	0.000		
AT2G36980	usa	4	0	3	0.358	1.127	0.758	-0.949	0
AT2G41360	usa	1	0	2	0.440	0.314	0.440	0.842	
AT2G43680	usa	0	0	1	0.000	0.000	0.000		
AT2G44990	usa	0	0	1	0.000	0.000	0.000		
AT2G46550	usa	0	0	1	0.000	0.000	0.000		
AT2G47430	usa	2	0	4	0.647	0.564	1.026	1.924	
AT3G10340	usa	1	0	2	0.523	0.291	0.523	1.505	
AT3G13290	usa	0	0	1	0.000	0.000	0.000		
AT3G20820	usa	1	0	2	0.667	0.545	0.667	1.633	
AT3G23590	usa	0	0	1	0.000	0.000	0.000		
AT3G44530	usa	1	0	2	0.366	0.291	0.366	0.488	
AT3G48690	usa	4	0	6	0.848	1.325	1.939	1.633	0
AT3G50740	usa	4	0	5	0.800	1.127	1.705	1.484	0
AT3G51570	usa	0	0	1	0.000	0.000	0.000		
AT3G54720	usa	1	0	2	0.416	0.274	0.416	0.895	
AT3G55060	usa	0	0	1	0.000	0.000	0.000		
AT3G62890	usa	0	0	1	0.000	0.000	0.000		
AT4G00030	usa	0	0	1	0.000	0.000	0.000		
AT4G02390	usa	1	0	2	0.533	0.353	0.533	1.303	
AT4G04350	usa	2	1	3	0.600	0.707	0.733	0.120	
AT4G08170	usa	0	0	1	0.000	0.000	0.000		
AT4G08840	usa	2	0	4	0.778	0.581	1.026	1.866	
AT4G10340	usa	10	1	6	0.889	3.535	3.511	-0.030	0
AT4G12030	usa	28	0	2	0.533	9.898	14.933	2.436	0
AT4G14180	usa	0	0	1	0.000	0.000	0.000		
AT4G14190	usa	11	0	3	0.392	3.198	2.301	-1.024	0
AT4G14210	usa	2	1	3	0.511	0.707	0.556	-0.691	
AT4G16280	usa	1	0	2	0.467	0.353	0.467	0.820	
AT4G18040	usa	6	0	3	0.622	2.121	2.844	1.411	1
AT4G18520	usa	1	0	2	0.529	0.291	0.529	1.548	
AT4G20410	usa	0	0	1	0.000	0.000	0.000		
AT4G22720	usa	11	2	5	0.800	3.888	4.556	0.768	0
AT4G25540	usa	0	0	1	0.000	0.000	0.000		
AT4G28395	usa	0	0	1	0.000	0.000	0.000		
AT4G30950	usa	6	1	3	0.600	2.121	2.867	1.455	0

locus	pop.	S	η_1	N_h	H_e	θ_w	θ_π	D	ρ
AT4G33250	usa	3	1	4	0.733	1.060	0.911	-0.507	0
AT4G36060	usa	1	0	2	0.533	0.353	0.533	1.303	
AT4G38160	usa	0	0	1	0.000	0.000	0.000		
AT4G38630	usa	2	0	2	0.533	0.707	1.067	1.641	
AT4G39680	usa	1	1	2	0.100	0.282	0.100	-1.164	
AT4G40080	usa	0	0	1	0.000	0.000	0.000		
AT5G04190	usa	3	0	3	0.394	0.823	0.788	-0.110	0
AT5G20280	usa	0	0	1	0.000	0.000	0.000		
AT5G41920	usa	0	0	1	0.000	0.000	0.000		
AT5G43670	usa	10	2	5	0.683	3.014	4.275	1.561	0
AT5G48100	usa	9	0	10	0.918	2.469	4.004	2.085	2
AT5G51670	usa	0	0	1	0.000	0.000	0.000		
AT5G53020	usa	0	0	1	0.000	0.000	0.000		
AT5G66280	usa	1	0	2	0.455	0.274	0.455	1.142	
