# Supplementary figures and images for: Patterns of Polymorphism and Demographic History in Natural Populations of Arabidopsis lyrata
Source: PLoS One. 2008 Jun 11;3(6):e2411. doi: 10.1371/journal.pone.0002411 (PMC2408968; doi:10.1371/journal.pone.0002411)

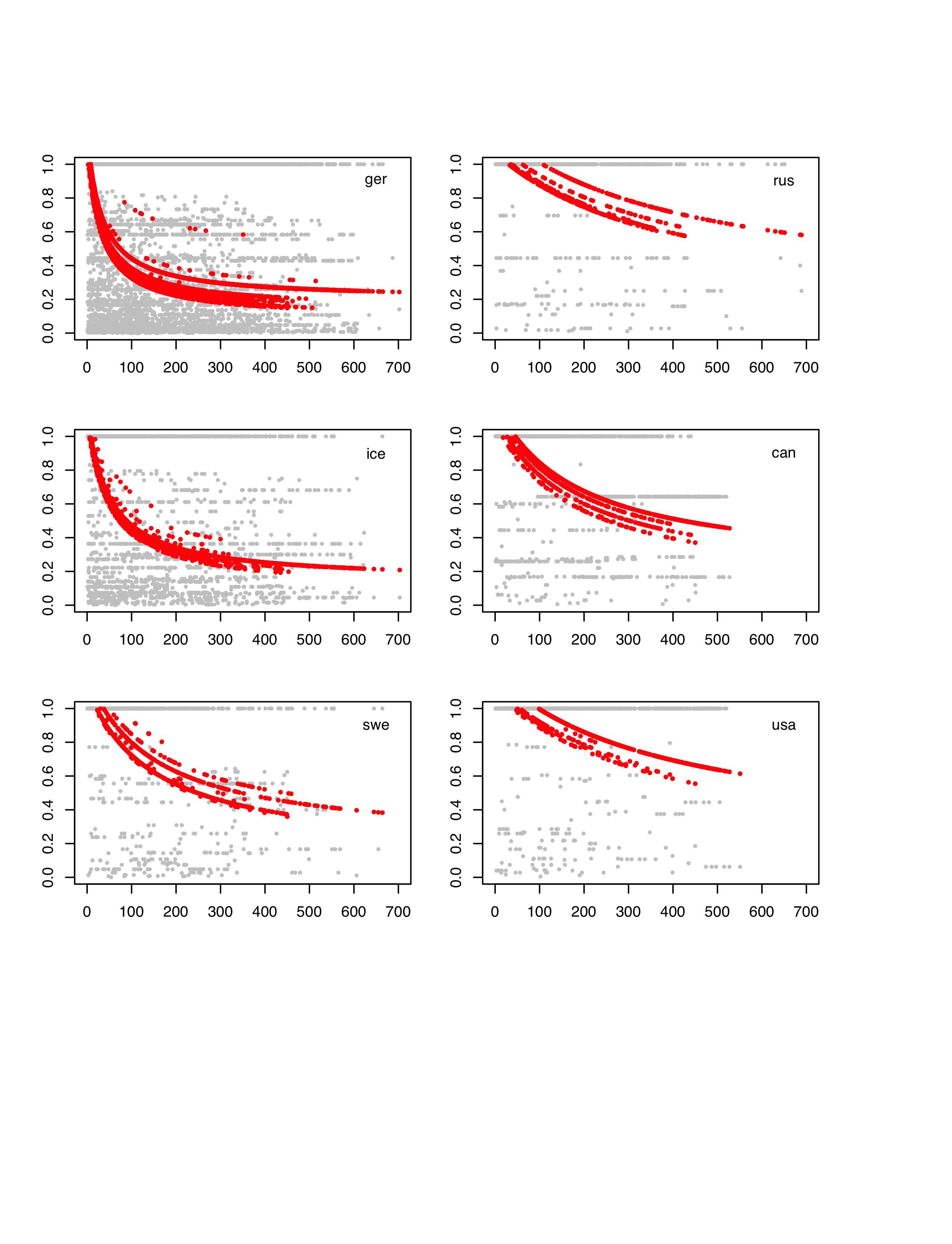

Supplement: Figure S1 — Decline in linkage disequilibrium over distance. Plotted is a lowess regression fit of intralocus r2 against distance for all SNPs in all loci. (0.59 MB TIF) [file pone.0002411.s004.tif]
